# Supplementary material for: Pneumococcus uses COMMD2 to alter host cellular immunity
Source: Nat Commun. 2026 May 27;17:7733. doi: 10.1038/s41467-026-72852-4 (PMC13434726; doi:10.1038/s41467-026-72852-4)
Supplement: Supplementary file 1 [file 41467_2026_72852_MOESM1_ESM.pdf]

SUPPLEMENTARY INFORMATION

A

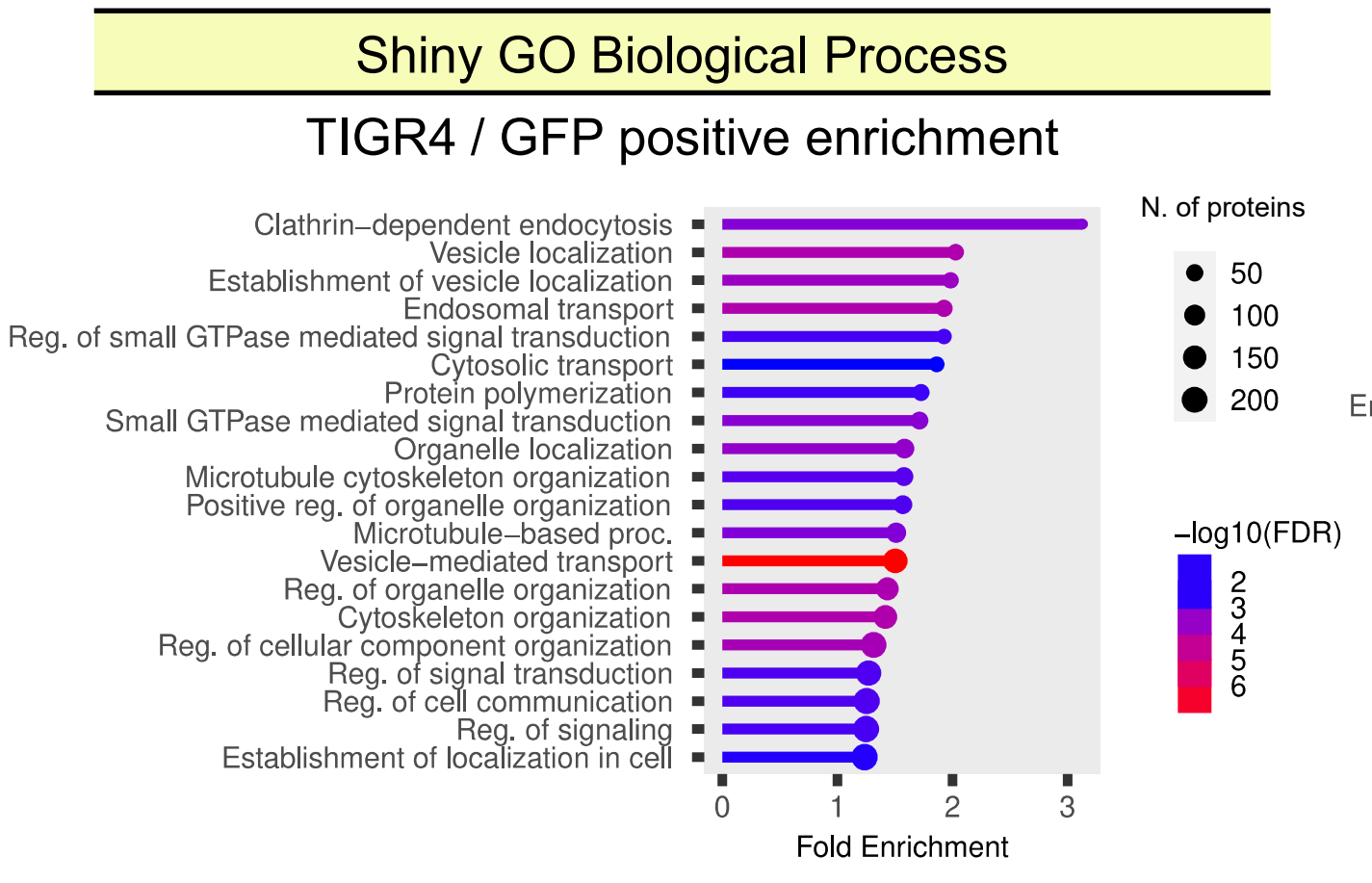

B

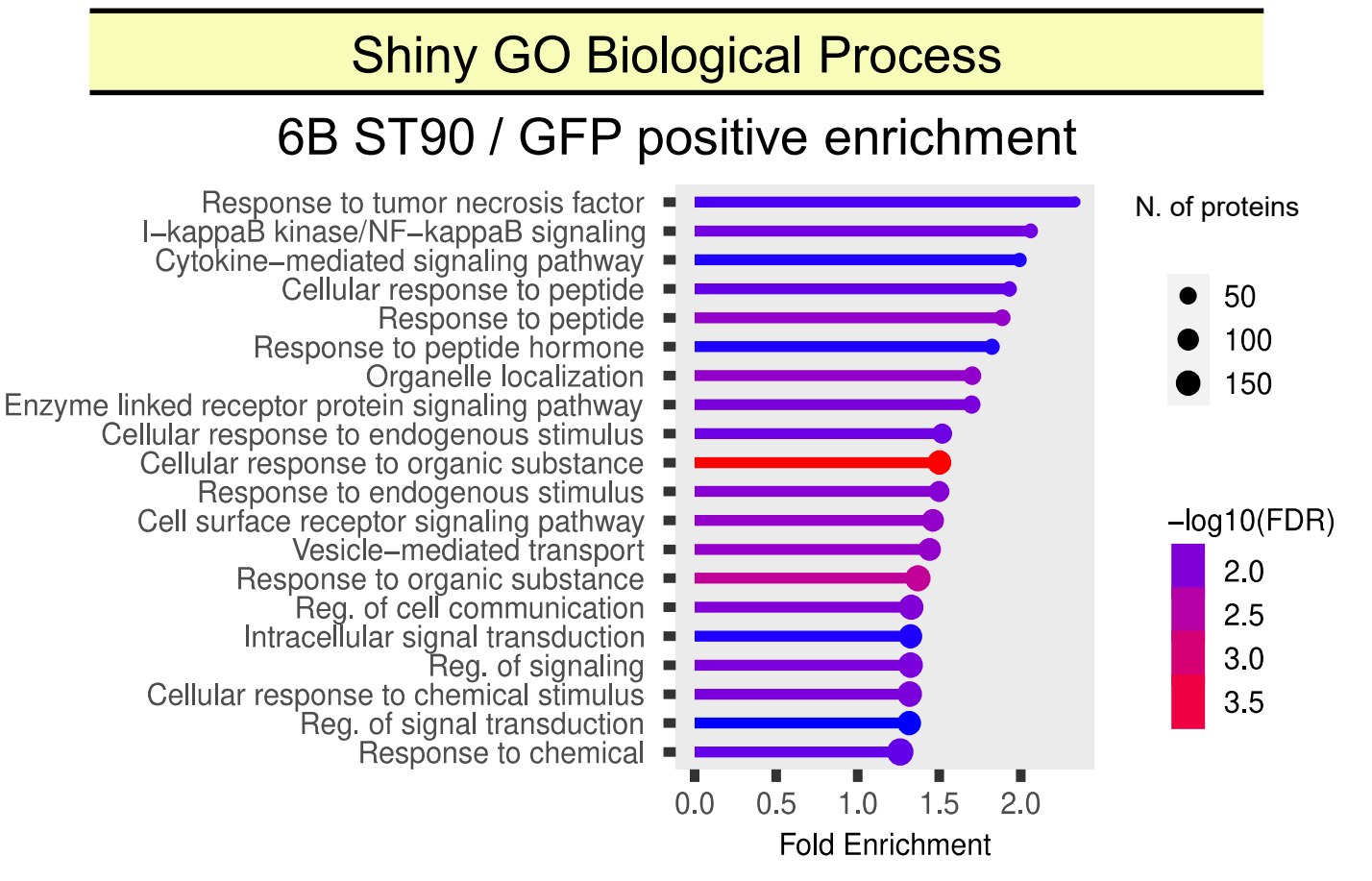

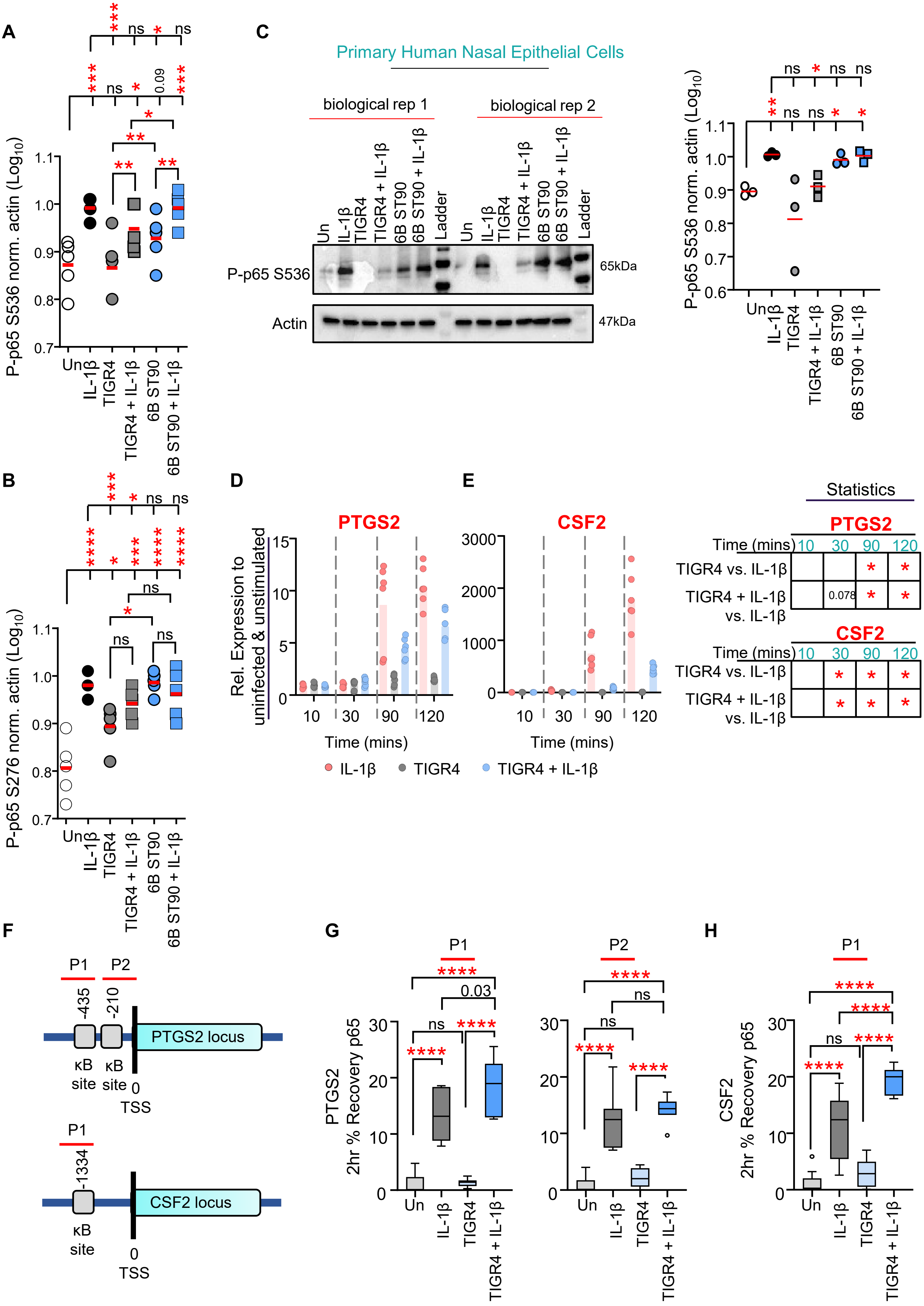

A
B
C
D
E
F
G
H
Primary Human Nasal Epithelial Cells
biological rep 1
biological rep 2
Un
IL-1β
TIGR4
TIGR4 + IL-1β
6B ST90
6B ST90 + IL-1β
Ladder
P-p65 S536
Actin
65kDa
47kDa
P-p65 S536 norm. actin (Log10)
P-p65 S276 norm. actin (Log10)
ns
0.09
PTGS2
CSF2
Rel. Expression to uninfected & unstimulated
Time (mins)
IL-1β
TIGR4
TIGR4 + IL-1β
Statistics
Time (mins)
TIGR4 vs. IL-1β
TIGR4 + IL-1β vs. IL-1β
0.078
P1
P2
-435
-210
κB site
0
TSS
PTGS2 locus
-1334
CSF2 locus
PTGS2 2hr % Recovery p65
CSF2 2hr % Recovery p65
0.03

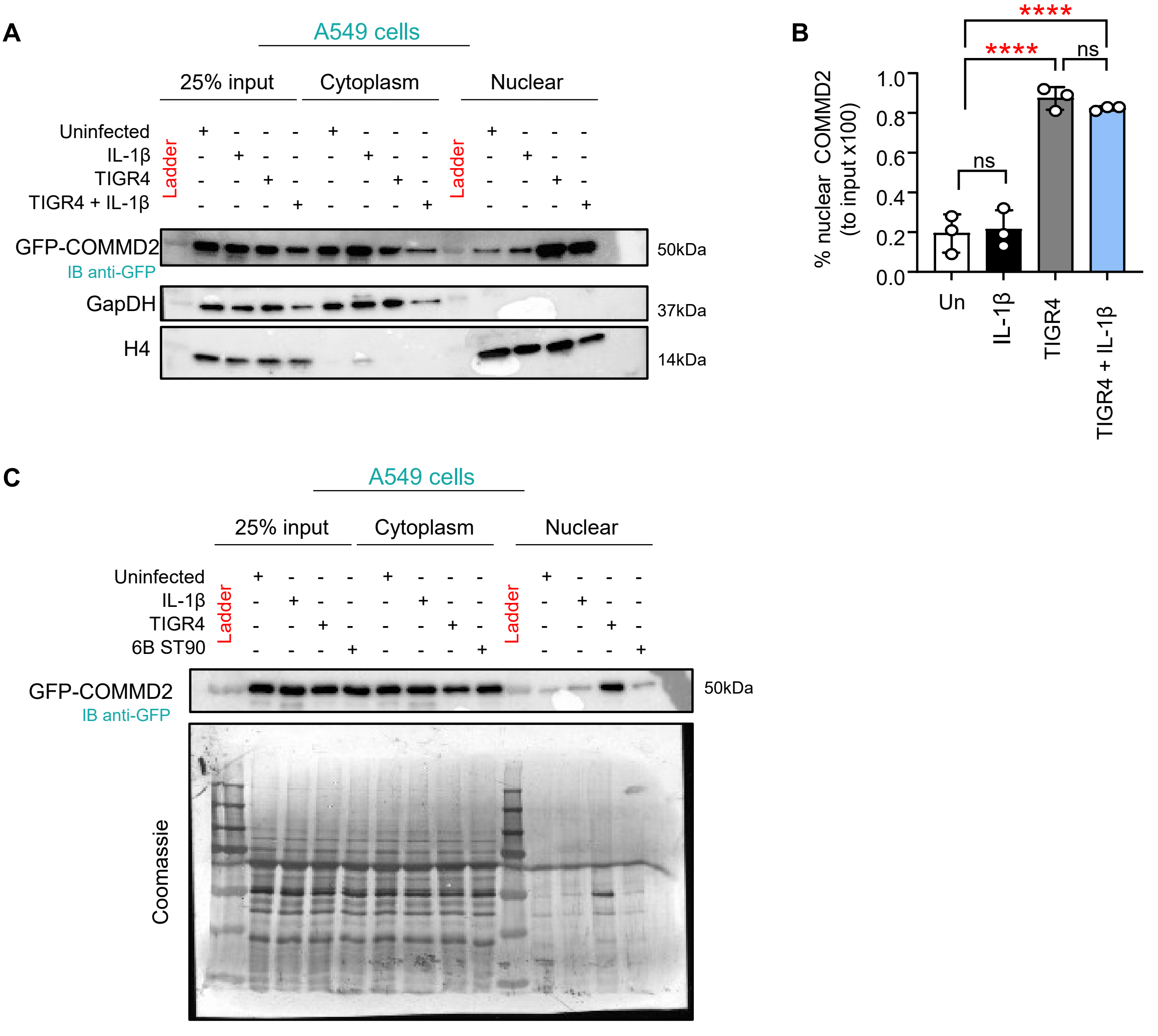
A
A549 cells
25% input
Cytoplasm
Nuclear
Uninfected
IL-1β
TIGR4
TIGR4 + IL-1β
Ladder
GFP-COMMD2
IB anti-GFP
GapDH
H4
50kDa
37kDa
14kDa
B
% nuclear COMMD2 (to input x100)
1.0
0.8
0.6
0.4
0.2
0.0
****
ns
Un
IL-1β
TIGR4
TIGR4 + IL-1β
C
A549 cells
25% input
Cytoplasm
Nuclear
Uninfected
IL-1β
TIGR4
6B ST90
Ladder
GFP-COMMD2
IB anti-GFP
50kDa
Coomassie

A

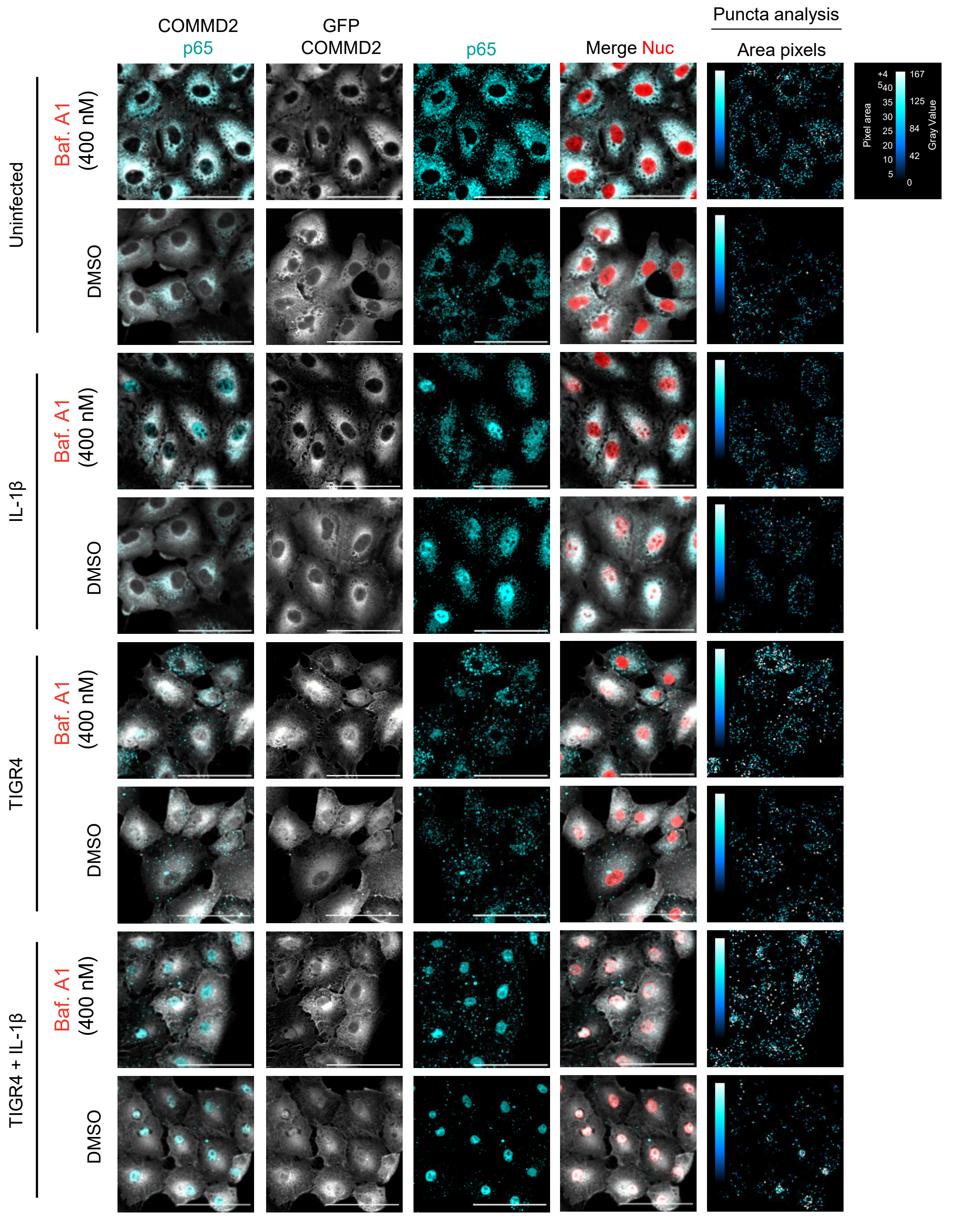

COMMD2
p65
GFP
COMMD2
p65
Merge Nuc
Puncta analysis
Area pixels
Uninfected
IL-1β
TIGR4
TIGR4 + IL-1β
Baf. A1
(400 nM)
DMSO
Pixel area
Gray Value
167
125
84
42
0

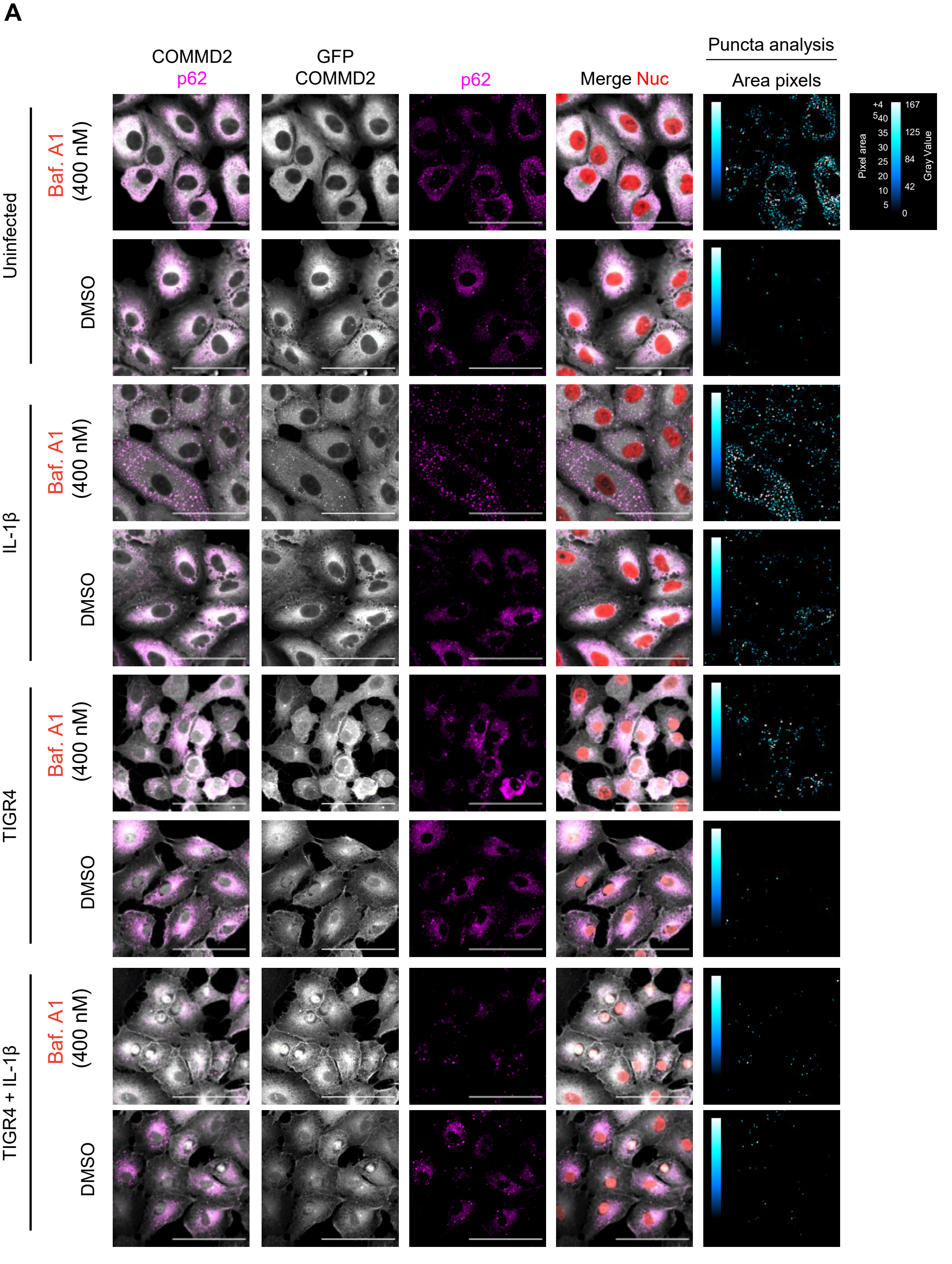
A
COMMD2
p62
GFP
COMMD2
p62
Merge Nuc
Puncta analysis
Area pixels
Uninfected
Baf. A1
(400 nM)
DMSO
IL-1β
Baf. A1
(400 nM)
DMSO
TIGR4
Baf. A1
(400 nM)
DMSO
TIGR4 + IL-1β
Baf. A1
(400 nM)
DMSO
Pixel area
+4
540
35
30
25
20
10
5
167
125
84
42
0
Gray Value

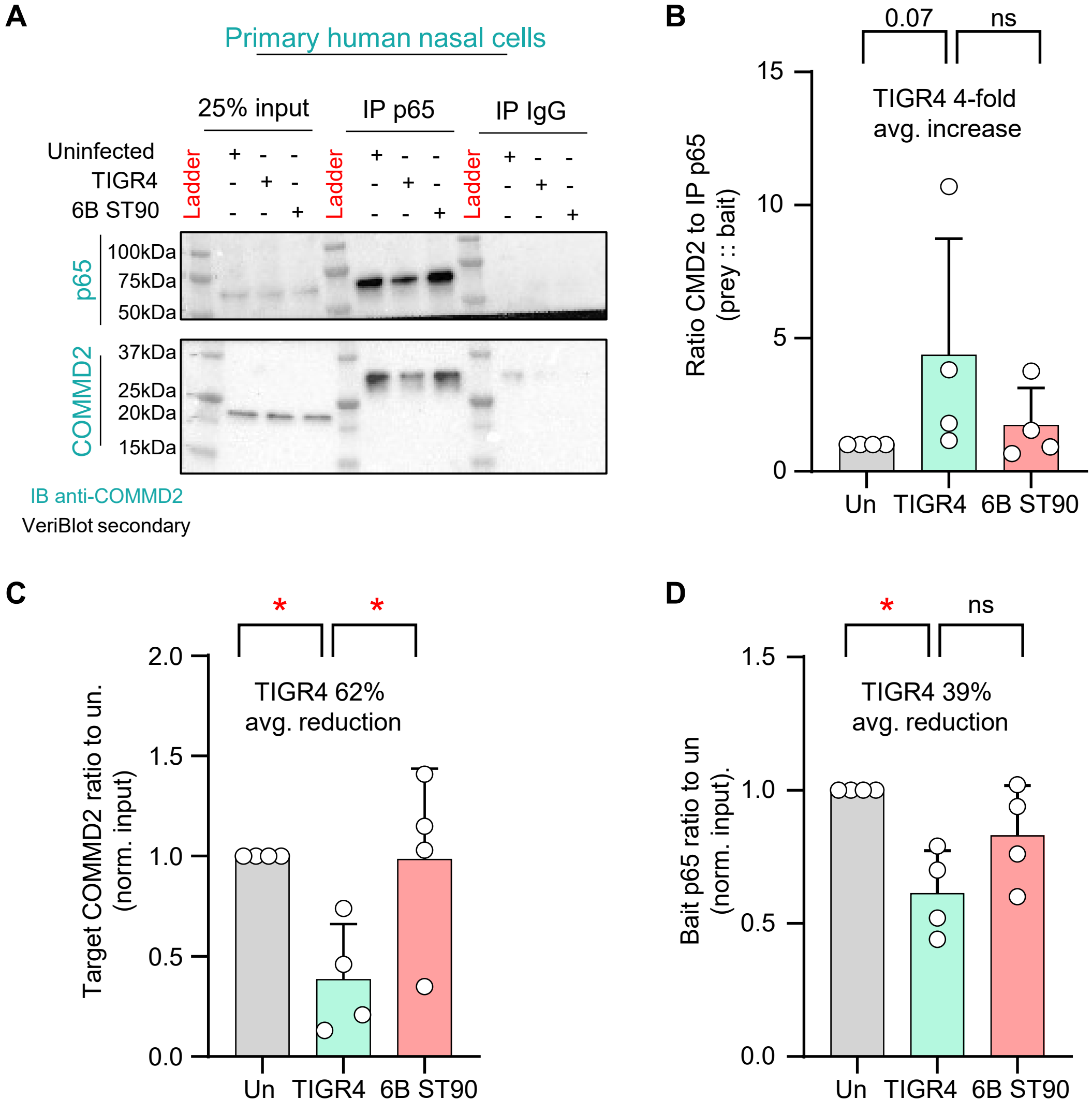
A
Primary human nasal cells
25% input
IP p65
IP IgG
Uninfected
TIGR4
6B ST90
Ladder
+ - -
- + -
- - +
p65
100kDa
75kDa
50kDa
COMMD2
37kDa
25kDa
20kDa
15kDa
IB anti-COMMD2
VeriBlot secondary
B
0.07
ns
TIGR4 4-fold
avg. increase
Ratio CMD2 to IP p65
(prey :: bait)
15
10
5
0
Un
TIGR4
6B ST90
C
*
*
TIGR4 62%
avg. reduction
Target COMMD2 ratio to un.
(norm. input)
2.0
1.5
1.0
0.5
0.0
Un
TIGR4
6B ST90
D
*
ns
TIGR4 39%
avg. reduction
Bait p65 ratio to un
(norm. input).
1.5
1.0
0.5
0.0
Un
TIGR4
6B ST90

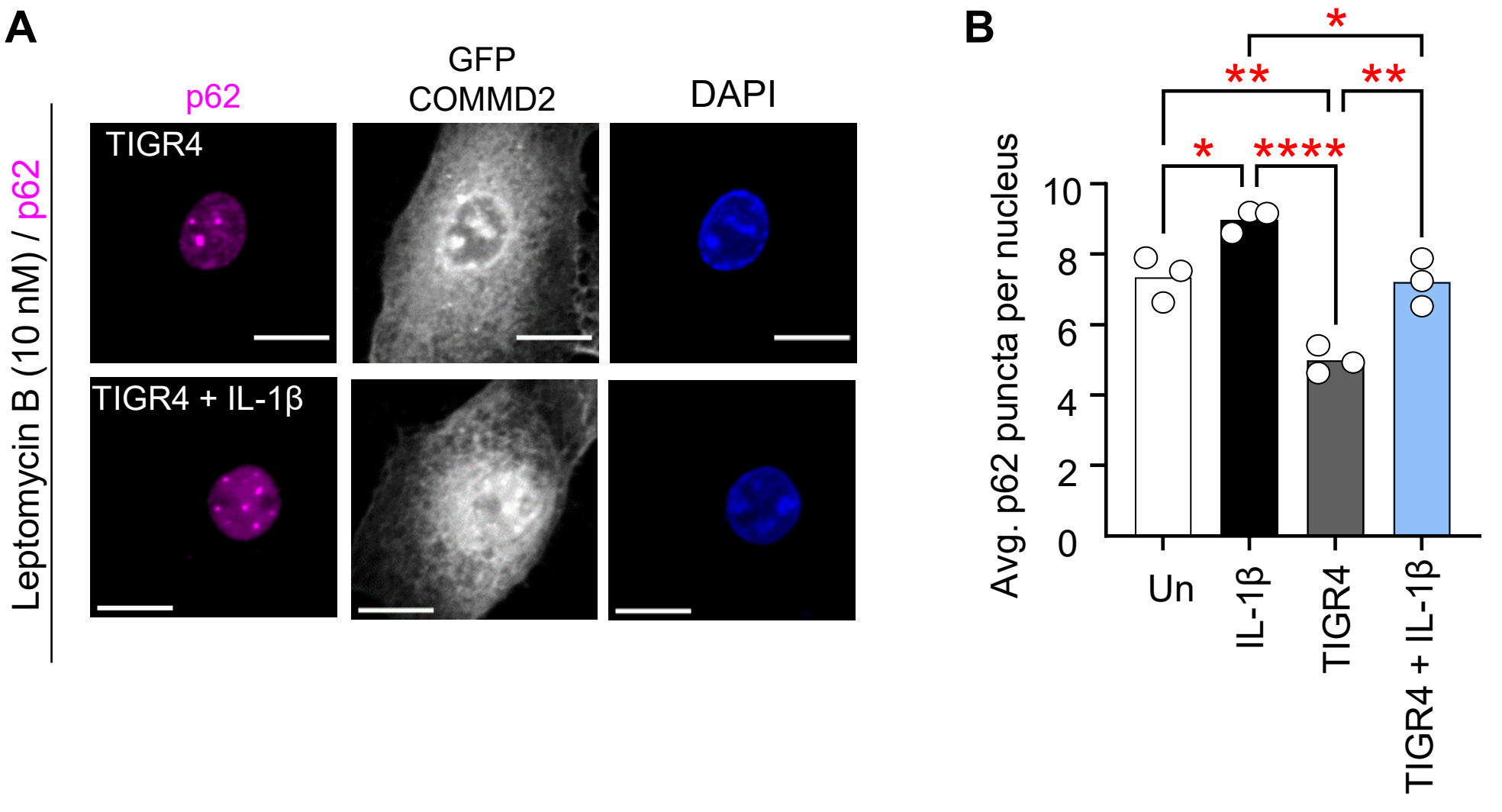

A
p62
GFP
COMMD2
DAPI
TIGR4
TIGR4 + IL-1β
Leptomycin B (10 nM) / p62
B
Avg. p62 puncta per nucleus
10
8
6
4
2
0
Un
IL-1β
TIGR4
TIGR4 + IL-1β
*
**
**
*
****

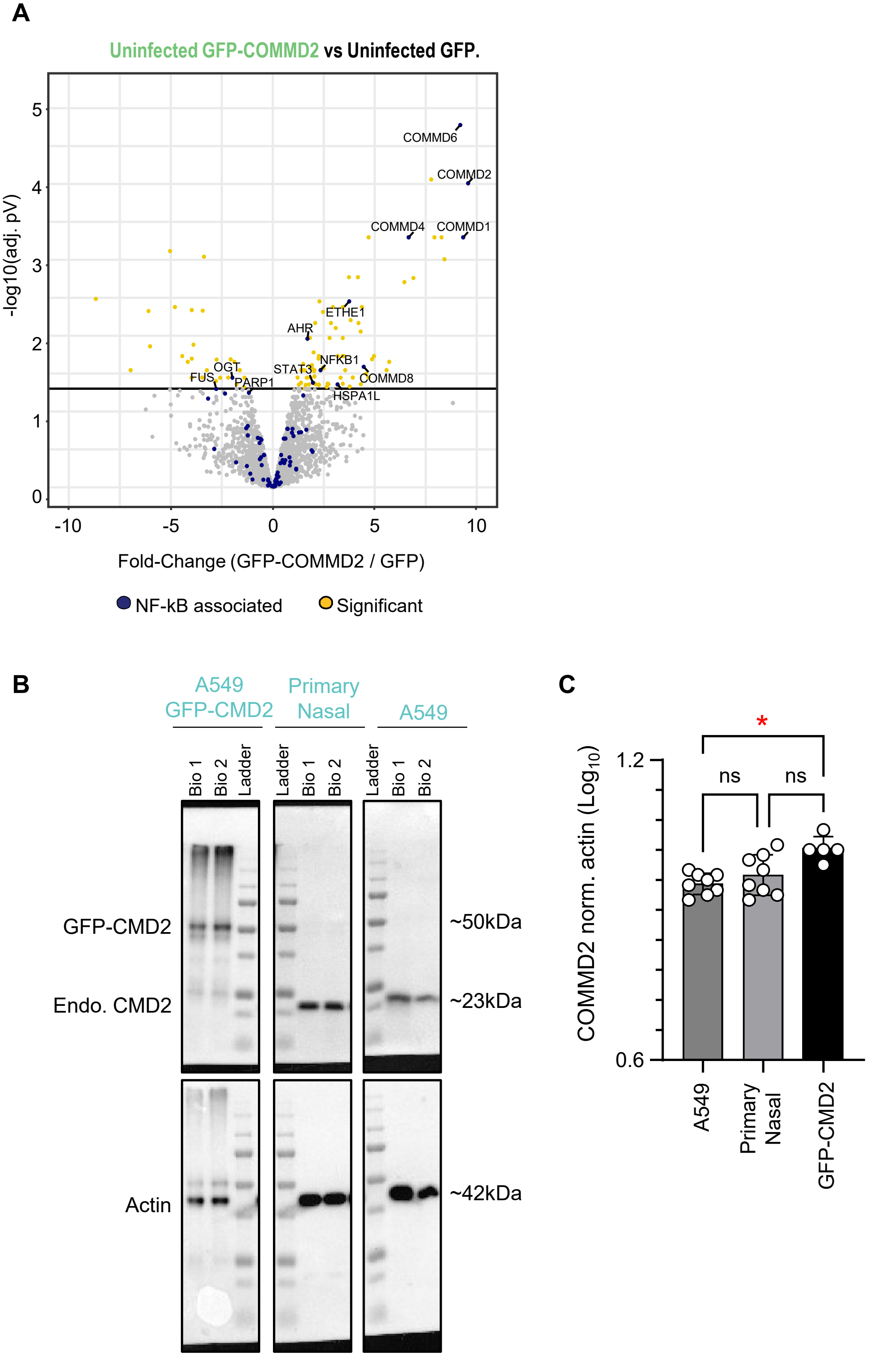
A
Uninfected GFP-COMMD2 vs Uninfected GFP.
COMMD6
COMMD2
COMMD4
COMMD1
ETHE1
AHR
NFKB1
STAT3
OGT
FUS
PARP1
COMMD8
HSPA1L
-log10(adj. pV)
Fold-Change (GFP-COMMD2 / GFP)
NF-kB associated
Significant
B
A549 GFP-CMD2
Primary Nasal
A549
Bio 1
Bio 2
Ladder
GFP-CMD2
Endo. CMD2
Actin
~50kDa
~23kDa
~42kDa
C
COMMD2 norm. actin (Log10)
ns
ns
*
A549
Primary Nasal
GFP-CMD2

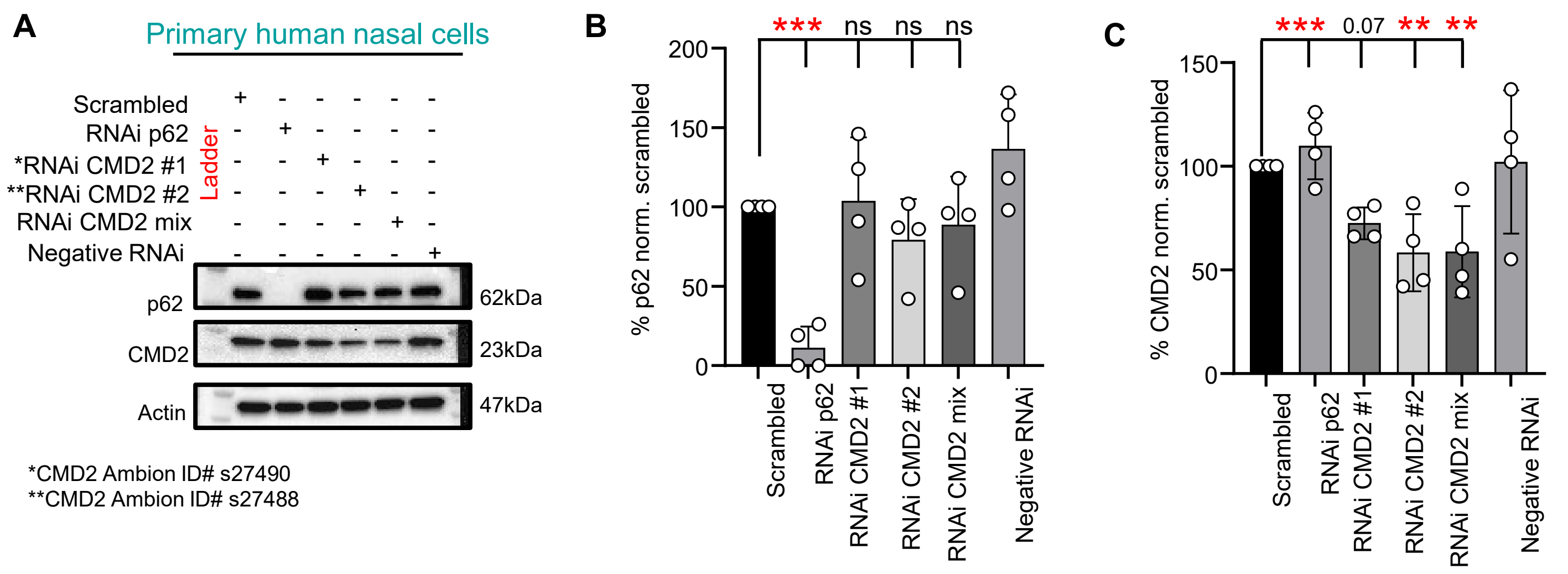

Primary human nasal cells

D — To Scr uninfected

	IL-1β	TIGR4	6B ST90	TIGR4 + IL-1β	6B ST90 + IL-1β
CCL3	1.977	1.343	0.473	1.917	2.063
CCL20	2.863	1.023	1.133	2.743	2.770
CXCL1	1.600	0.427	0.580	1.893	1.890
CXCL10	3.113	0.950	1.263	2.793	3.123
ICAM1	3.680	1.527	1.967	2.510	2.490
IL1ra (IL1RN)	0.357	1.270	1.703	1.480	1.763
IL-8	2.723	1.460	1.543	3.090	3.390
IL11	3.287	0.990	1.657	3.473	3.493
IL-13	0.867	0.440	0	1.490	1.157
IL-18	1.023	0.347	0.300	1.317	0.913
MMP2	4.107	2.763	3.140	4.217	4.243
MMP9	0.663	0.400	0.393	0.893	1.103
SELE	1.100	0.677	0.503	1.280	1.313
SLPI	0.333	0.273	0.197	0.427	0.310
SCGB1A	0.307	0.520	0.553	0.740	0.683
CX3CL1	1.810	1.310	1.473	1.930	1.863
CXCL2	4.113	1.577	1.547	4.653	4.613
CSF2	2.220	0.533	0.507	2.510	2.440
IFNA1	0.433	0.313	0.070	0.467	0.470
IL-6	5.320	1.940	1.797	6.157	5.943
IL-10	0.923	0.643	0.397	1.083	1.387
IL-17C	4.763	0.910	0.097	4.850	6.480
MMP1	0.890	0.477	0.647	0.920	0.807
MMP3	2.147	1.400	1.463	2.233	2.397
PTX3	2.030	0.863	0.340	2.280	2.240
SELP	2.917	2.397	2.383	3.217	2.973
TNFa	5.110	0.447	1.450	6.007	5.580

0 2 4 6

4hr Avg. Luminex Analyte Expression to uninfected Scr control (log_2)

E — RNAi p62

	Un.	IL-1β	TIGR4	6B ST90	TIGR4 + IL-1β	6B ST90 + IL-1β
CCL3	0.260	-0.037	0.040	1.210	0.143	-0.017
CCL20	0.073	0.137	0.700	0.620	0.657	0.200
CXCL1	0.137	0.113	0.173	0.087	-0.130	-0.200
CXCL10	3.423	2.447	3.347	3.067	2.777	2.357
ICAM1	0.933	-0.790	0.237	0.287	1.100	0.757
IL1ra (IL1RN)	0.153	0.200	0.357	0.437	0.503	0.513
IL-8	0.587	0.250	0.370	0.163	0.283	-0.283
IL11	0.483	0.103	1.040	0.687	0.093	0.033
IL-13	0.660	0.037	0.113	0.973	0	0.087
IL-18	0.077	0.023	0.320	-0.177	-0.223	0.967
MMP2	1.460	0.083	0.273	0.100	0.010	-0.070
MMP9	0	0.403	-0.400	0.303	0.237	-0.263
SELE	0.033	0.017	-0.090	0	0.413	0.120
SLPI	0.093	0.200	0.090	0.303	0.300	0.320
SCGB1A	0.207	0.280	0.063	0.217	0.127	0.100
CX3CL1	1.267	0.100	0.263	0.200	-0.013	0.020
CXCL2	0.087	-0.130	-0.013	0.130	-0.163	-0.130
CSF2	0.120	-0.077	0.380	0.093	-0.063	0.083
IFNA1	0.017	-0.067	-0.100	0.180	-0.033	-0.260
IL-6	0.077	-0.467	-0.147	-0.290	-0.450	-0.553
IL-10	0	0.710	-0.483	0.063	0.613	0.323
IL-17C	1.477	-1.073	-0.367	0.080	2.320	-0.737
MMP1	0.340	0.257	0.447	0.570	0.493	0.497
MMP3	0.600	-0.083	0.303	0.200	0.023	-0.307
PTX3	0.263	0.693	0.427	0.950	0.317	0.453
SELP	1.747	0.190	0.040	0.087	0.187	-0.070
TNFa	0.387	-0.527	1.200	0.080	-0.453	-0.263

Significance to Scr

	Un.	IL-1β	TIGR4	6B ST90	TIGR4 + IL-1β	6B ST90 + IL-1β
CCL3	0.1420	0.7760	0.6940	0.3637	0.3870	0.8420
CCL20	0.8780	0.6810	0.1160	0.0044	0.0070	0.3800
CXCL1	0.5980	0.5560	0.2270	0.4627	0.4890	0.0380
CXCL10	0.0060	0.0010	0.0020	0.0004	0.0020	0.0020
ICAM1	0.3740	0.2410	0.8040	0.8613	0.3910	0.5940
IL1ra (IL1RN)	0.7580	0.3880	0.1360	0.1112	0.0360	0.0620
IL-8	0.0470	0.6170	0.2370	0.6062	0.5020	0.1870
IL11	0.1410	0.5380	0.1800	0.3629	0.6230	0.7520
IL-13	0.3740	0.9630	0.7140	0.1902	1.0000	0.8100
IL-18	0.8740	0.9550	0.1300	0.6342	0.5400	0.5020
MMP2	0.4550	0.3210	0.5470	0.1755	0.9340	0.1170
MMP9		0.5440	0.3740	0.1298	0.2030	0.3740
SELE	0.3740	0.9500	0.3740		0.3520	0.4250
SLPI	0.5310	0.2440	0.3880	0.0226	0.0920	0.0930
SCGB1A	0.4320	0.2200	0.4350	0.2467	0.4610	0.5550
CX3CL1	0.1210	0.4880	0.5900	0.3068	0.9280	0.8180
CXCL2	0.8540	0.3120	0.9200	0.4146	0.1670	0.0230
CSF2	0.1190	0.8210	0.2730	0.3739	0.6730	0.6150
IFNA1	0.9350	0.7480	0.3740	0.5329	0.8480	0.1780
IL-6	0.8450	0.0070	0.3030	0.4049	0.0620	0.0400
IL-10		0.4030	0.6100	0.3739	0.2670	0.3190
IL-17C	0.1950	0.1560	0.8460	0.3739	0	0.3280
MMP1	0.0530	0.3240	0.1420	0.0003	0.1060	0.0870
MMP3	0.1980	0.7950	0.3740	0.5122	0.9000	0.1130
PTX3	0.1190	0.1420	0.5190	0.1228	0.2890	0.2140
SELP	0.1240	0.5410	0.8950	0.5686	0.6530	0.1580
TNFa	0.1380	0.0860	0.3960	0.5309	0.1140	0.0240

Not calculated | Borderline | pV ≤0.05

-1 0 1 2 3

4hr Avg. Luminex Analyte Expression to individual Scr control (log_2)

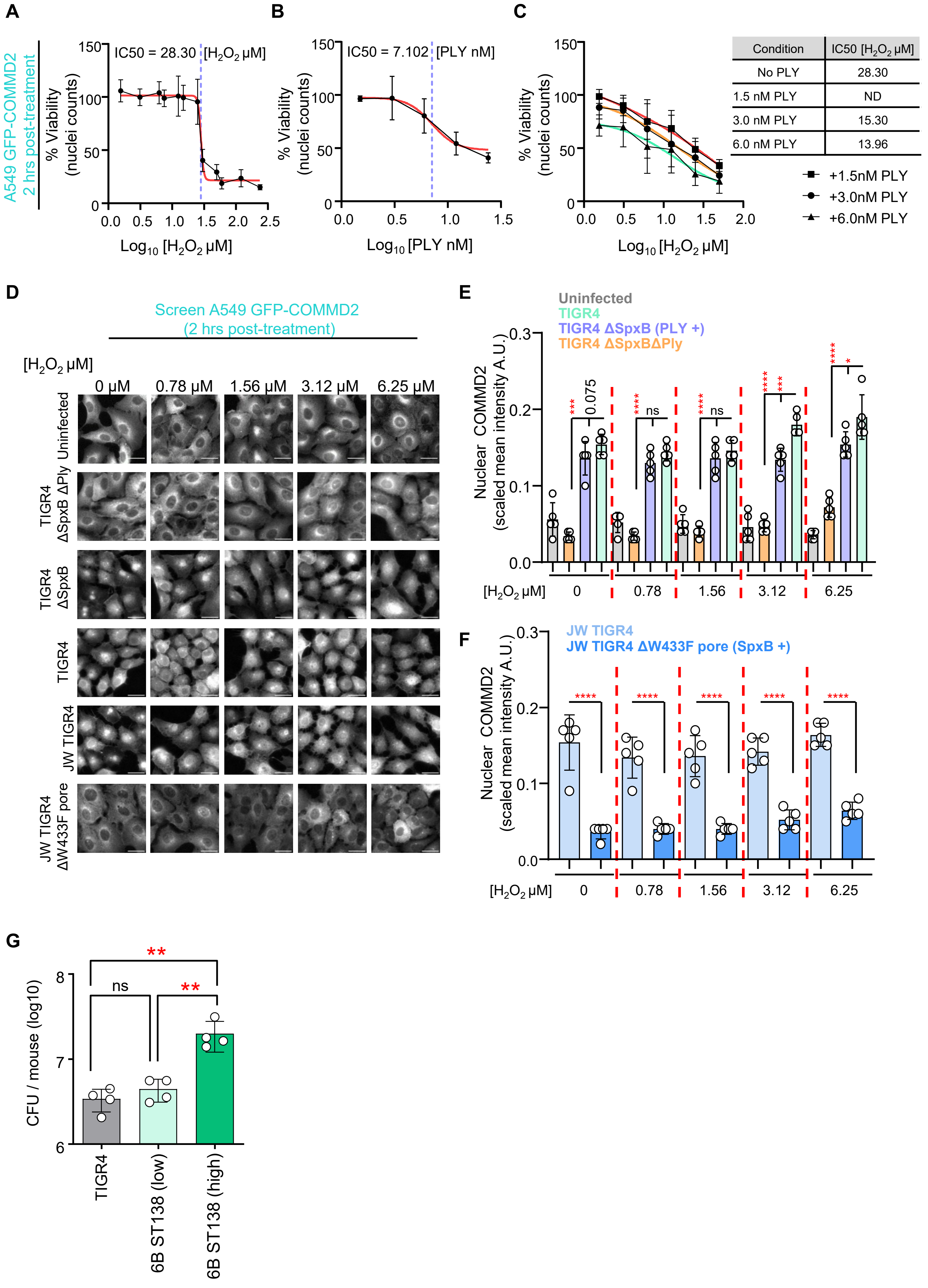
A
A549 GFP-COMMD2
2 hrs post-treatment
% Viability (nuclei counts)
IC50 = 28.30
[H2O2 µM]
Log10 [H2O2 µM]
B
IC50 = 7.102
[PLY nM]
Log10 [PLY nM]
C
Condition
IC50 [H2O2 µM]
No PLY
28.30
1.5 nM PLY
ND
3.0 nM PLY
15.30
6.0 nM PLY
13.96
+1.5nM PLY
+3.0nM PLY
+6.0nM PLY
Log10 [H2O2 µM]
D
Screen A549 GFP-COMMD2
(2 hrs post-treatment)
[H2O2 µM]
0 µM
0.78 µM
1.56 µM
3.12 µM
6.25 µM
Uninfected
TIGR4 ΔSpxB ΔPly
TIGR4 ΔSpxB
TIGR4
JW TIGR4
JW TIGR4 ΔW433F pore
E
Uninfected
TIGR4
TIGR4 ΔSpxB (PLY +)
TIGR4 ΔSpxBΔPly
Nuclear COMMD2 (scaled mean intensity A.U.)
[H2O2 µM]
F
JW TIGR4
JW TIGR4 ΔW433F pore (SpxB +)
Nuclear COMMD2 (scaled mean intensity A.U.)
[H2O2 µM]
G
CFU / mouse (log10)
ns
TIGR4
6B ST138 (low)
6B ST138 (high)

Sup. Figure 1: Individual GO biological process enrichment for TIGR4 and 6B ST90 p65 interactomes. A-B) TIGR4 or 6B ST90 enrichment over GFP. Lollipop graph indicating FDR by color scale and number of proteins by lollipop size.

Sup. Figure 2: TIGR4 drives disparity in NF-kB gene expression and chromatin binding. A-B) phosphorylated p65 at Serine 536 or Serine 276 to actin. C) Immunoblot of primary nasal whole cell lysates 2 hrs post-challenge with either IL-1β (10 ng/ml), TIGR4 (MOI 20) or 6B ST90 (MOI 20) (± IL-1β; 10 ng/ml) probed for phosphorylated p65 at Serine 536 and actin (n=3 biological). A – C) Dot blot with mean (red line). One-way ANOVA with Dunnett's multiple comparison post-hoc test. Intergroup comparison paired T-Test. $**P \leq 0.01$, $***P \leq 0.001$, $****P \leq 0.0001$. Full blots provided in Supplementary Information 1. D-E) Inflammatory gene transcription profiles by RT-qPCR for PTGS2 & CSF2 of A549 cells over a 2 hr time course with either IL-1β or TIGR4 (± IL-1β 10 ng/ml; MOI 20). Graphed as the relative expression of each indicated transcript to matched uninfected/unstimulated control per time point (n = 3 biological replicates; 2 technicals per replicate). Displayed as a dot plot with each data point and a bar representing the mean. Statistics table reporting significance calculated by unpaired T.Test at each time point indicated. * = significant. F) Schematic representation of PTGS2 or CSF2 promoter with ChIP-qPCR primer locations and NF-κB sites denoted as predicted by Alibaba2-curated eukaryotic transcription factor DNA-binding motifs from the TRANSFAC database [68]. G-H) 10μg chromatin input from A549 cells either untreated (light gray), IL-1β treated (10 ng/ml; dark gray) or 2 hrs post-challenge with TIGR4 (± IL-1β; 10 ng/ml; MOI 20; light blue or dark blue respectively). ChIP-qPCR represented as % recovery against input of p65 at indicated NF-κB sites. Tukey box and whisker plot with defined box boundaries being the upper and lower interquartile range (IQR), 'whiskers' (fences) being ± 1.5 times IQR and the median depicted by the middle solid line (n=3 biological replicates with 2 or 3 technicals per replicate). One-way ANOVA comparing all means with Tukey's multiple comparison post-hoc test. ns=not significant, $****P \leq 0.0001$. Source data are provided as a Source Data file.

Sup. Figure 3: TIGR4 challenge drives COMMD2 nuclear localization. Cell fractions from a stable A549 GFP-COMMD2 cell line 2 hrs post-challenge with either IL-1β (10 ng/ml), TIGR4 (MOI 20) or 6B ST90 (MOI 20). A) Representative immunoblot of cell fractions probed for GFP (COMMD2) enrichment across cellular compartments with GapDH (cytoplasm control) or H4 (nuclear control). B) Quantified nuclear COMMD2 (n = 3 biological). Bar graph with dots representing experiments. One-way ANOVA with Tukey's multiple comparison post-hoc test. ns = not significant, $****P \leq 0.0001$. C) Representative immunoblot of cell fractions and coomassie stained PVDF membrane. Source data are provided as a Source Data file.

Sup. Figure 4: Representative images of COMMD2 & p65 with Bafilomycin A1. Representative confocal microscopy images of COMMD2 (grey), p65 (cyan) and nucleus (red) of A549 cells pre-treated with Bafilomycin A1 (400nM) or DMSO control (n = 3 biological for p65, n = 6 for COMMD2). All conditions challenged with either IL-1β (10 ng/ml) or TIGR4 (± IL-1β 10 ng/ml; MOI 20). Puncta analysis image is of "Ice" LUT overlay of area (pixel) with color scale. Scale bar = 50μm. Source data are provided as a Source Data file.

Sup. Figure 5: Representative images of COMMD2 & p62 with Bafilomycin A1. Representative confocal microscopy images of COMMD2 (grey), p62 (pink) and nucleus (red) of A549 cells pre-treated with Bafilomycin A1 (400nM) or DMSO control (n = 3 biological for p62, n = 6 for COMMD2). All conditions challenged with either IL-1β (10 ng/ml) or TIGR4 (± IL-1β 10 ng/ml; MOI 20). Puncta analysis

image is of "Ice" LUT overlay of area (pixel) with color scale. Scale bar = 50µm. Source data are provided as a Source Data file.

Sup. Figure 6: TIGR4 induces endogenous COMMD2-p65 complex. A) Representative immunoblot of p65 immunoprecipitation from primary nasal cells 2 hr post-challenge with either TIGR4 or 6B ST90 (MOI 20; n= 4 biological replicates). B-D) Quantified ratio of p65 to COMMD2 (prey :: bait) normalized to uninfected (B), target COMMD2 ratio to uninfected normalized to input (C) and bait p65 ratio to uninfected normalized to input (D). Dot and bar graph. (B) Kruskal-Wallis test with Dunn's multiple comparison test. C-D) One-way ANOVA with Bonferroni's multiple comparison post-hoc test. *P ≤ 0.05. Source data are provided as a Source Data file.

Sup. Figure 7: CRM1 inhibition increases p62 puncta. A) Representative immunofluorescence confocal microscopy of stable A549 GFP-COMMD2 pretreated for 3 hrs with Leptomycin B (10 nM) prior to 2 hr challenge with either IL-1β (10 ng/ml) or TIGR4 ± IL-1β (MOI 20). Paraformaldehyde fixed cells stained for p62 (magenta) against GFP-COMMD2 (gray) and nuclei (DAPI; n=3 biological replicates; nuclei counts for Uninfected n=1041, IL-1β n=831, TIGR4 n=1164, TIGR4 + IL-1β n=1269). Scale bar = 10 µm. B) Nuclear p62 puncta average counts per cell. Graphed as mean with dots representing individual experiments. One-way ANOVA comparing all means with Tukey's multiple comparison post-hoc test. ns=not significant, *P ≤ 0.05, **P ≤ 0.01, ****P ≤ 0.0001. Source data are provided as a Source Data file.

Sup. Figure 8: GFP-COMMD2 vs. GFP volcano plot. A) Mass-spectrometry interactome (n=2 biological replicates per condition) of immunoprecipitated GFP-COMMD2 from a stable A549 GFP-COMMD2 cell line ($1x10^7$ cells total) or GFP A549 cell line control ($1x10^7$ cells total). Volcano plot of GFP-COMMD2 vs. GFP alone control. Identified interacting partners with known NF-kB p65 interaction in blue and significant targets in yellow. Lines represent -log10(pV) and fold-change cutoffs with targets of interest denoted. B) Representative immunoblot of COMMD2 levels from whole cell lysates across the A549 GFP-COMMD2 cell line (n=5 biological replicates), A549 parent cell line (n=8 biological replicates) and primary human nasal cells (n=8 biological replicates). C) Bar graph of COMMD2 normalized to actin, with Kruskal-Wallis test with Dunn's multiple comparison test. *P ≤ 0.05. Source data are provided as a Source Data file and proteomics data in Supplementary Data table 2.

Sup. Figure 9: RNAi efficiency in primary nasal cells and Luminex analyte analysis of p62 RNAi. A) Representative immunoblot of RNAi conditions targeting COMMD2 or p62 in primary human nasal epithelial cells from whole cell lysates at 48 hrs of RNAi (n = 4 biological replicates). PVDF membrane probed for either COMMD2 or p62 protein levels. B & C) Quantification of COMMD2 and p62 protein levels post RNAi. Dot blot with mean (bars) ± STD. One-way matched ANOVA with Dunnett's multiple comparison post-hoc test comparing RNAi conditions to scrambled control. ns = not significant, **P ≤ 0.01, ***P ≤ 0.001. D) Secreted analytes (Luminex) at 4 hrs from Scr control in primary nasal cells (n=3 biological replicates) challenged with either IL-1β (10 ng/ml), TIGR4 (MOI 20) ± IL-1β or 6B ST90 (MOI 20) ± IL-1β. E) Luminex of secreted analytes at 4 hrs from RNAi p62 in primary nasal cells normalized to scrambled controls (n=3 biological replicates). Cells were challenged with either IL-1β (10 ng/ml), TIGR4 (MOI 20) ± IL-1β or 6B ST90 (MOI 20) ± IL-1β. Individual p values shown in comparison to respected Scr control. Calculated by unpaired Two-tailed T. Test. Source data are provided as a Source Data file.

Sup. Figure 10: CMD2 nuclear translocation screen with pneumococcal mutants and hydrogen peroxide. A-C) Titration of hydrogen peroxide (A), purified Pneumolysin toxin (B), or their combination

(C) upon viability of the GFP-COMMD2 A549 stable cell line (n=4 biological replicates). Graphed as a dot ± STD of biological replicates for IC_{50} calculations by 4 parameter variable slope curve (red) with IC_{50} denoted in purple dashed line. Tabular comparison of IC_{50}. D) Representative deconvolution epifluorescence microscopy of A549 GFP-COMMD2 (gray) cells 2 hr post-challenge with either MOI 20 for each bacterial strain JW TIGR4 (wildtype), JW TIGR4 Δ*ply* W433F, TIGR4 (wildtype), TIGR4 *ΔspxB* or TIGR4 *ΔspxBΔply* under titrated hydrogen peroxide treatment (0 – 6.25µM). E-F) Graphed as Dot blot with mean (bars) ± STD. One-way matched ANOVA comparing selected columns with Bonferroni's multiple comparison post-hoc test. ns = not significant, *P ≤ 0.05, **P ≤ 0.01, ***P ≤ 0.001, ****P ≤ 0.0001. G) Inocula of intranasal mouse challenge shown as bar graph of mean ± STD. One-way ANOVA comparing all means with Tukey's multiple comparison post-hoc test. ns=not significant, **P ≤ 0.01. Source data are provided as a Source Data file.

Supplementary Data Table 1: Proteomics data for p65 interactome. All A549 GFP-p65 proteomics data. Sheet 1 contains the color code highlighting proteins of interest (yellow) alongside COMMD proteins, CCDC subunits and Retriever subunits (purple). Sheet 2 has all the statistical comparisons for TIGR4, 6B ST90, and negative (uninfected) GFP-p65 control. Sheet 3 is the quantified phosphosite peptides dataset.

Supplementary Data Table 2: Proteomics data for COMMD2 interactome. All A549 GFP-COMMD2 proteomics data. Sheet 1 contains the color code highlighting proteins of interest (yellow) alongside COMMD proteins, CCDC subunits and Retriever subunits (purple). Sheet 2 has all the statistical comparisons for TIGR4, 6B ST90, IL-1β, uninfected and negative (uninfected) GFP control. Sheet 3 is the quantified ubiquitinated peptides dataset.

Supplementary Data Table 3: Primers and bacterial strains. Data table with all primer information used for cloning and RT-qPCR alongside relevant information for all bacterial strains used in the study.

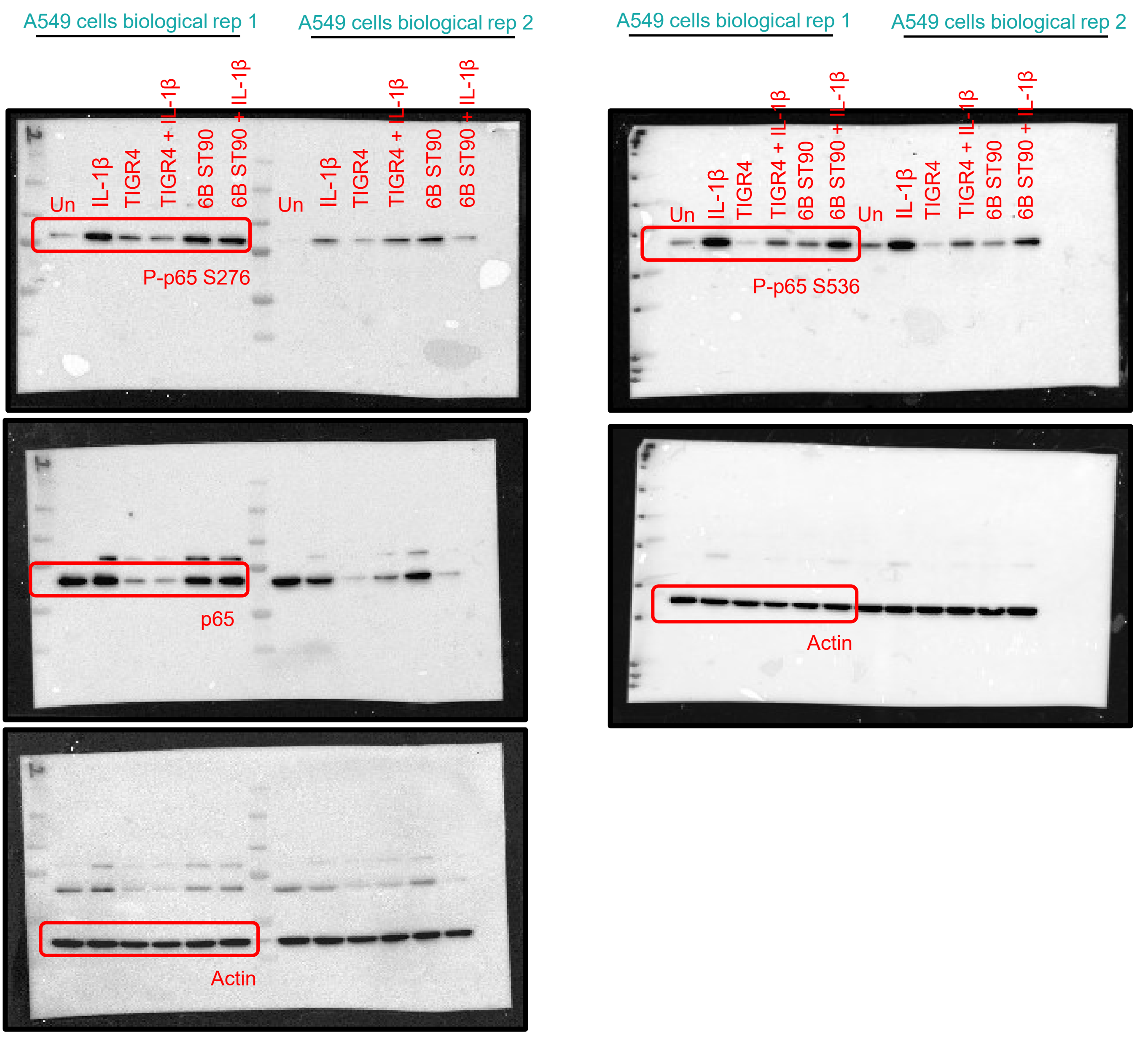

A549 cells biological rep 1
A549 cells biological rep 2
Un
IL-1β
TIGR4
TIGR4 + IL-1β
6B ST90
6B ST90 + IL-1β
P-p65 S276
p65
Actin
P-p65 S536

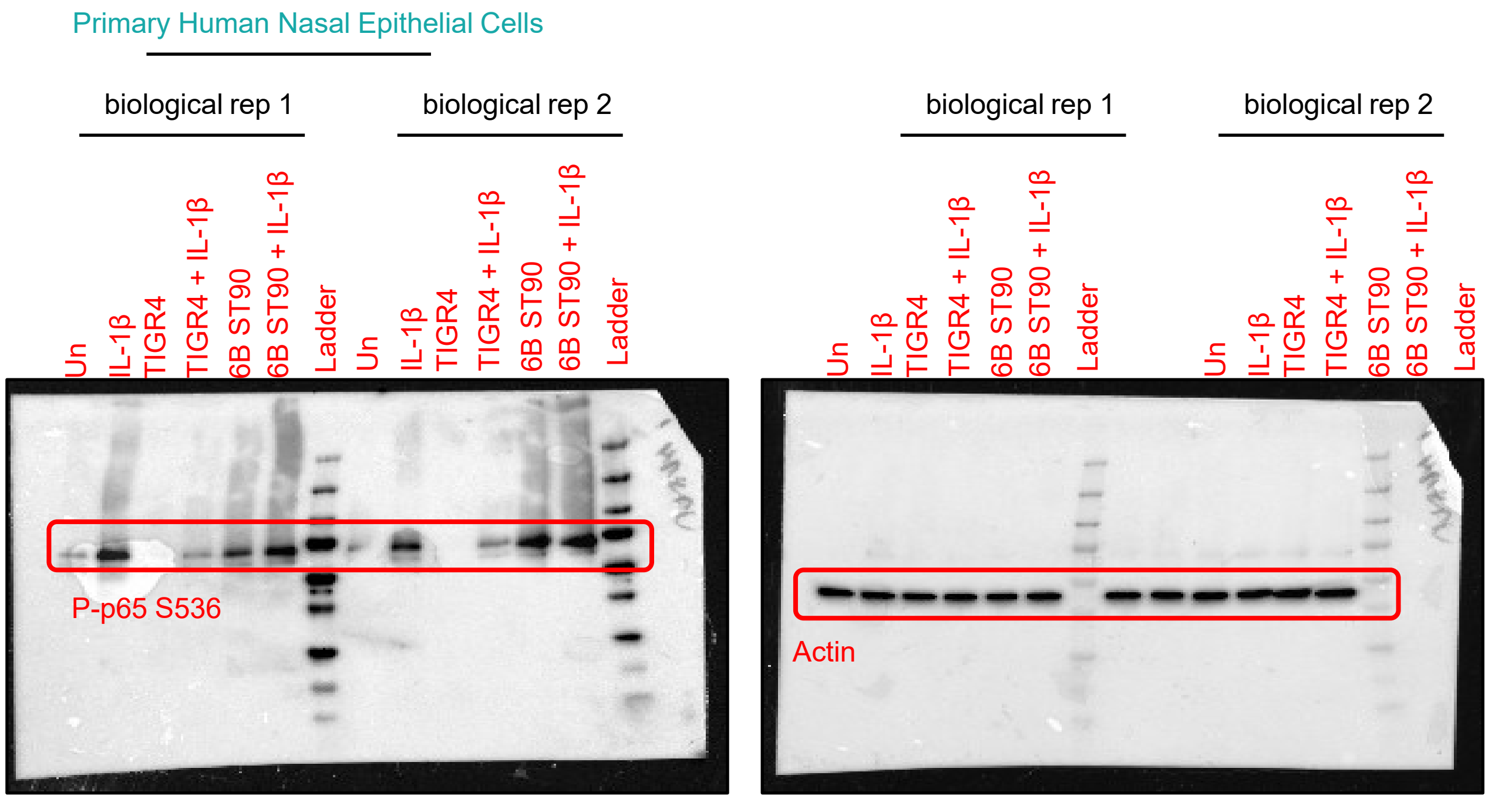

Primary Human Nasal Epithelial Cells
biological rep 1
biological rep 2
Un
IL-1β
TIGR4
TIGR4 + IL-1β
6B ST90
6B ST90 + IL-1β
Ladder
P-p65 S536
Actin

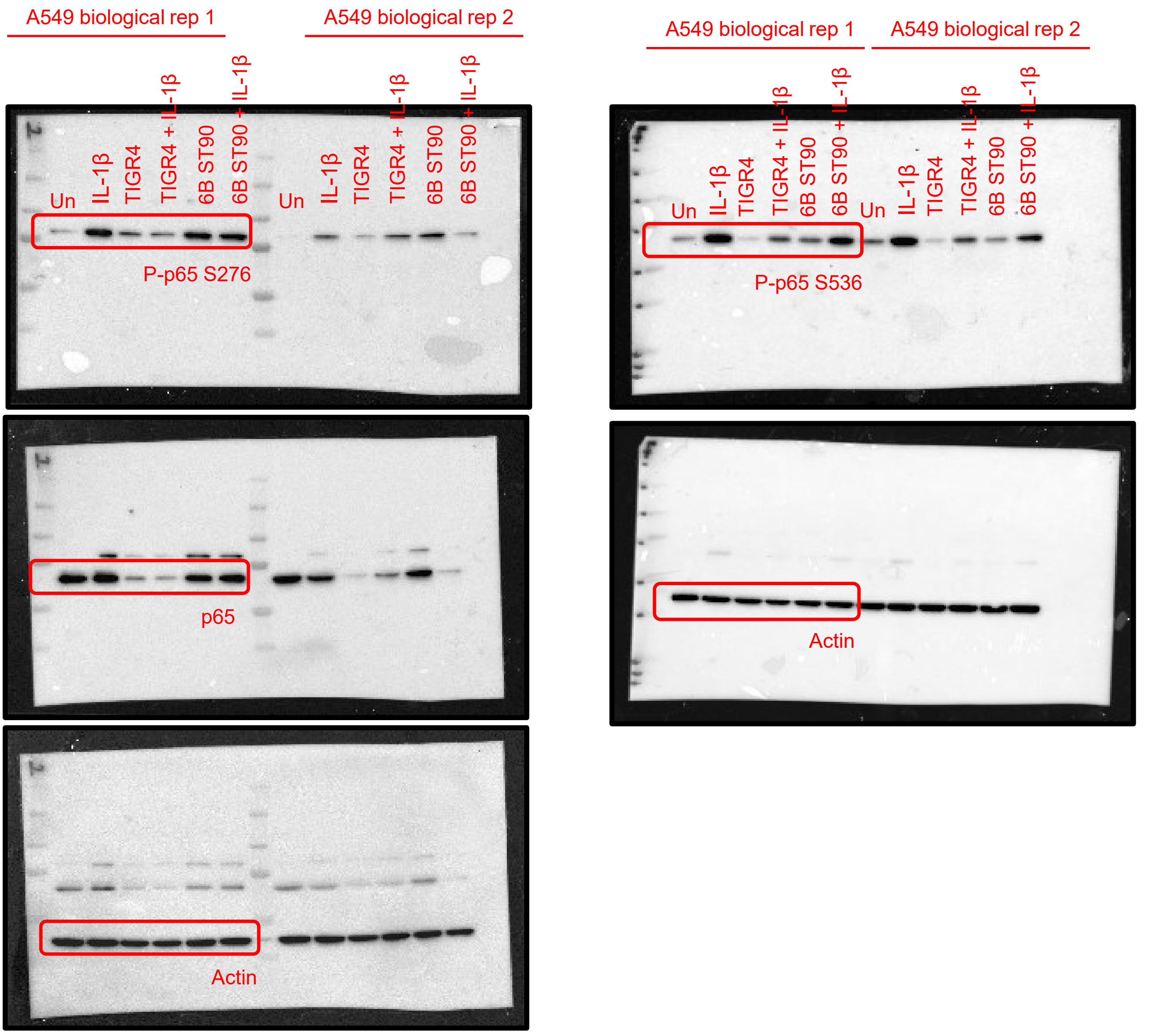
A549 biological rep 1
A549 biological rep 2
Un
IL-1β
TIGR4
TIGR4 + IL-1β
6B ST90
6B ST90 + IL-1β
P-p65 S276
p65
Actin
P-p65 S536
Actin

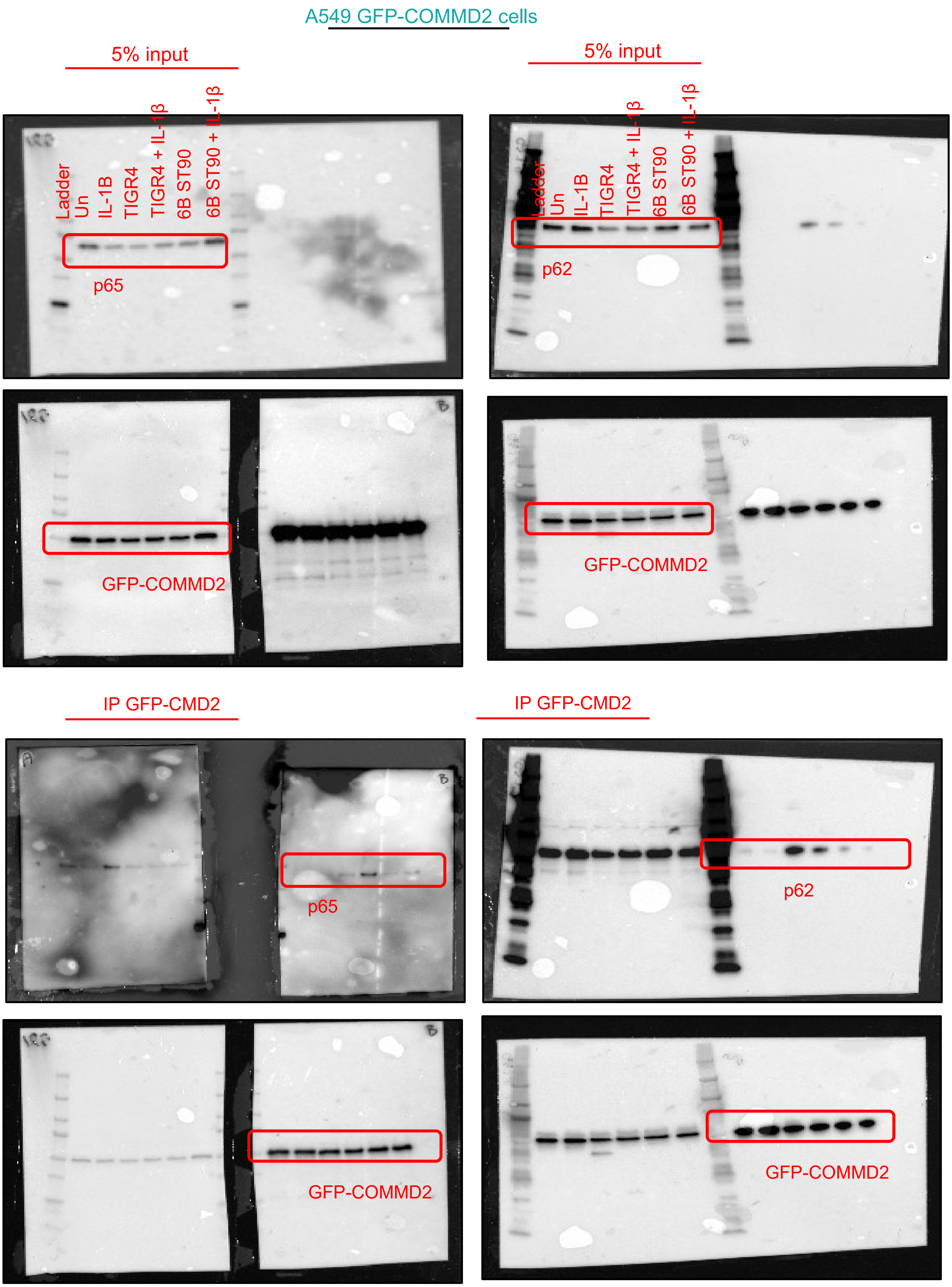
A549 GFP-COMMD2 cells
5% input
Ladder
Un
IL-1B
TIGR4
TIGR4 + IL-1β
6B ST90
6B ST90 + IL-1β
p65
5% input
Ladder
Un
IL-1B
TIGR4
TIGR4 + IL-1β
6B ST90
6B ST90 + IL-1β
p62
GFP-COMMD2
GFP-COMMD2
IP GFP-CMD2
IP GFP-CMD2
p65
p62
GFP-COMMD2
GFP-COMMD2

Primary human nasal epithelial cells

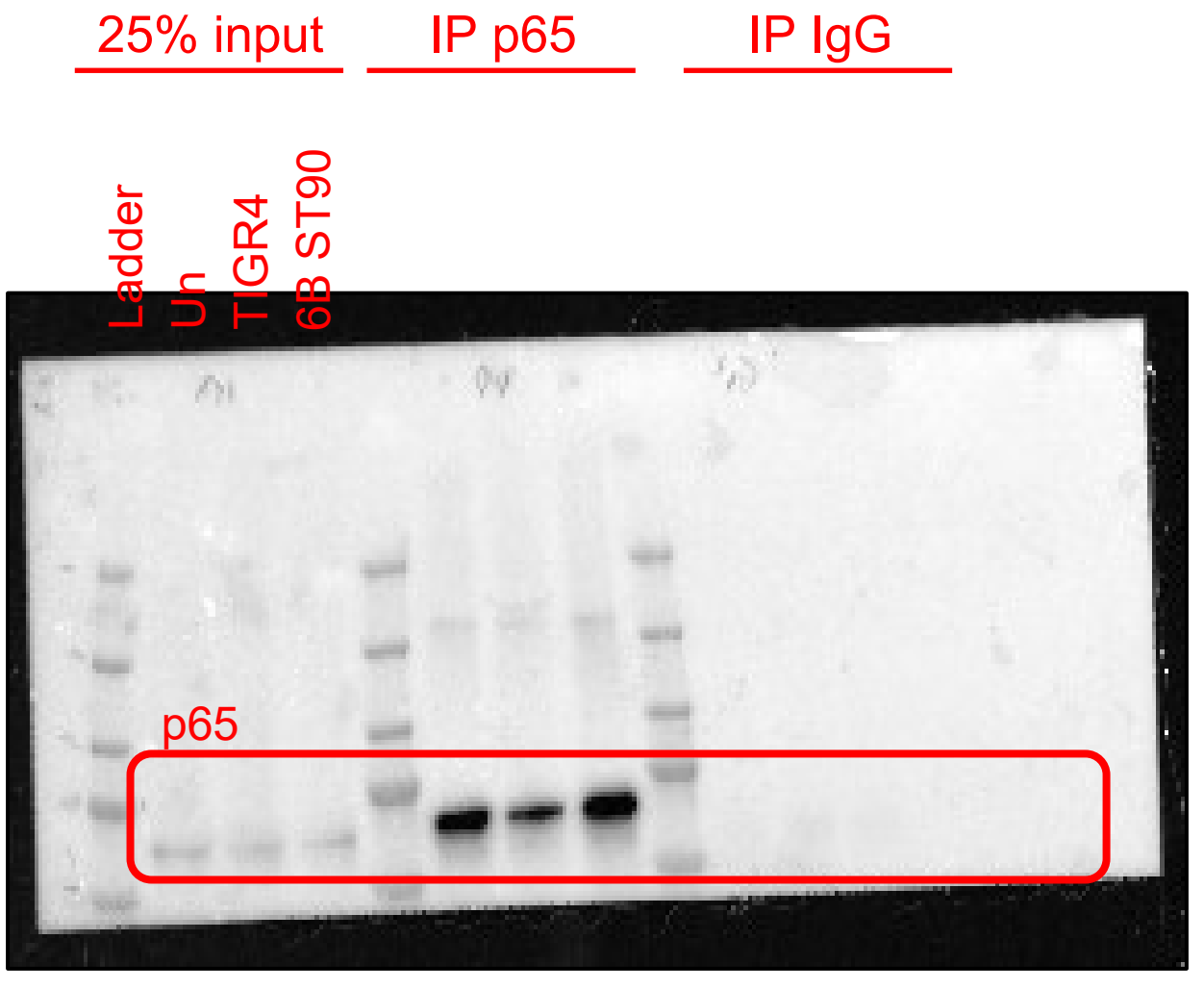

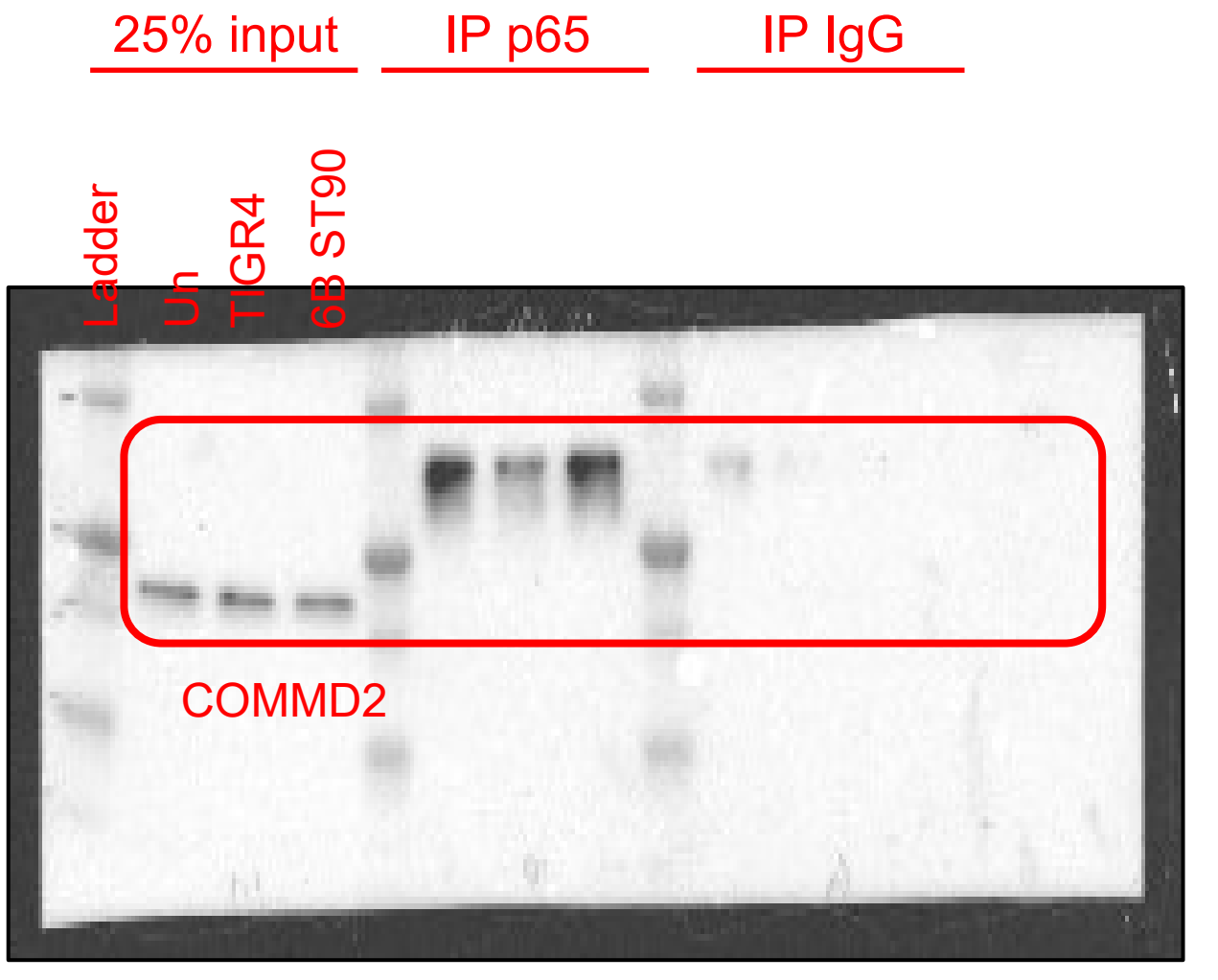

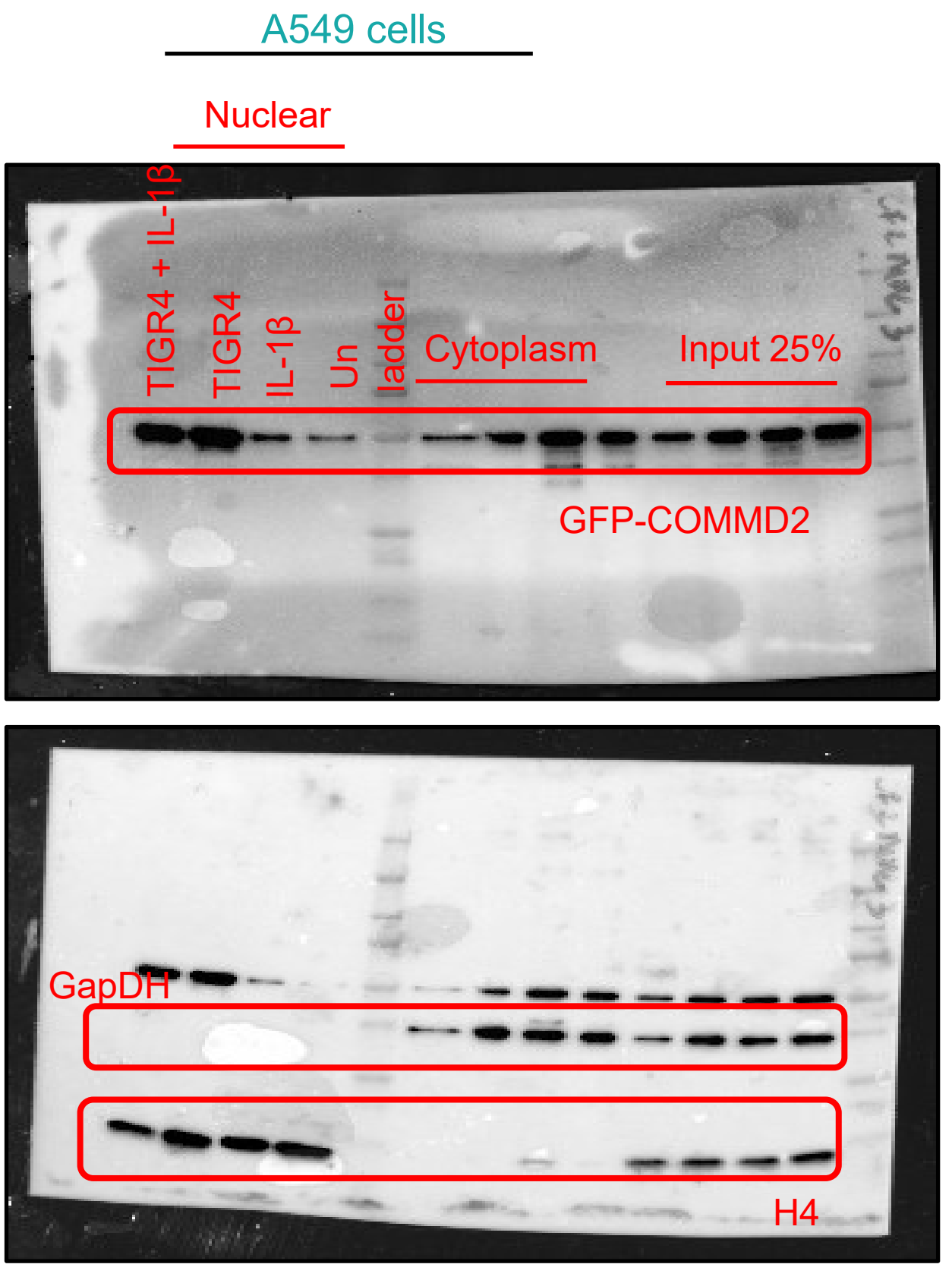
A549 cells
Nuclear
TIGR4 + IL-1β
TIGR4
IL-1β
Un
ladder
Cytoplasm
Input 25%
GFP-COMMD2
GapDH
H4

Primary human nasal cells

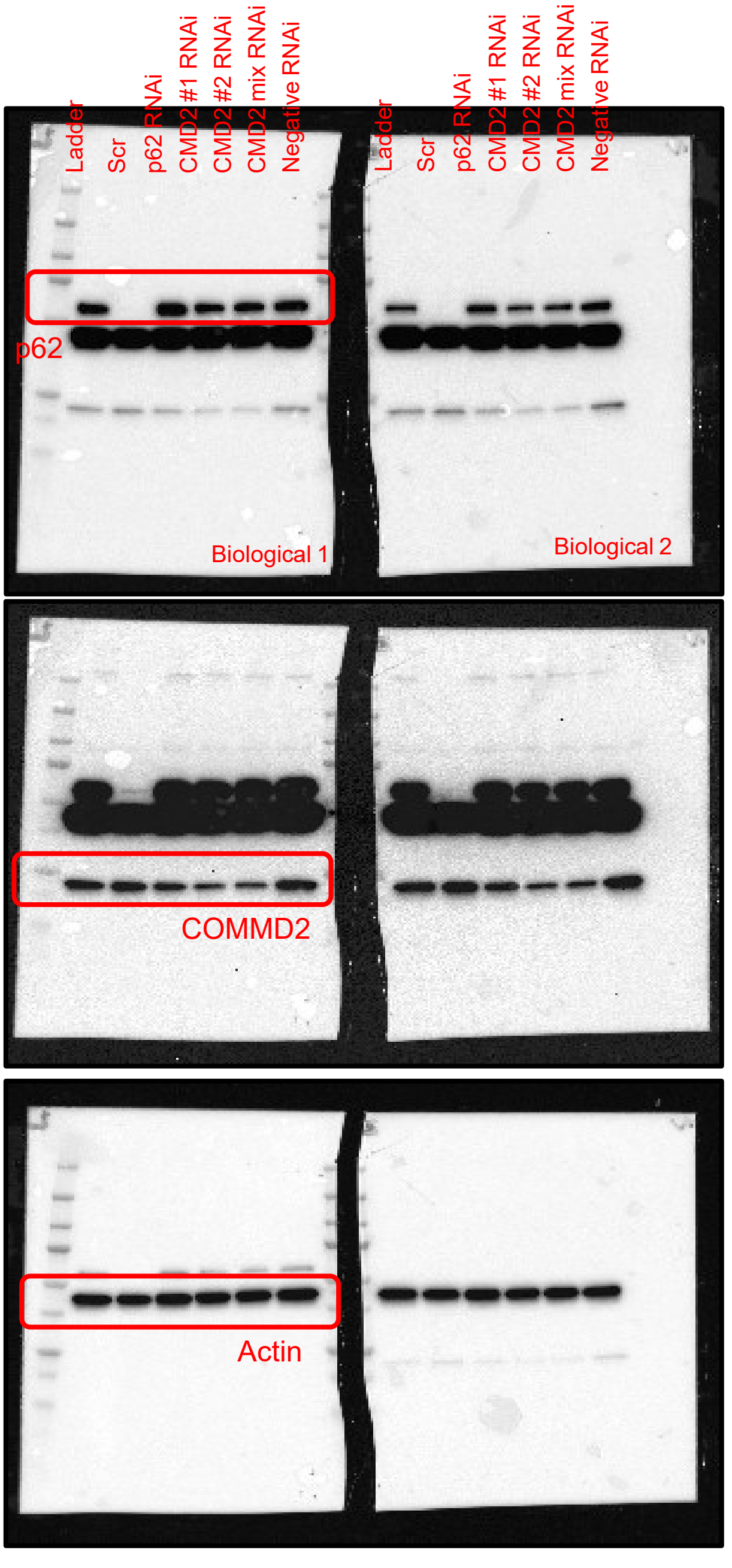

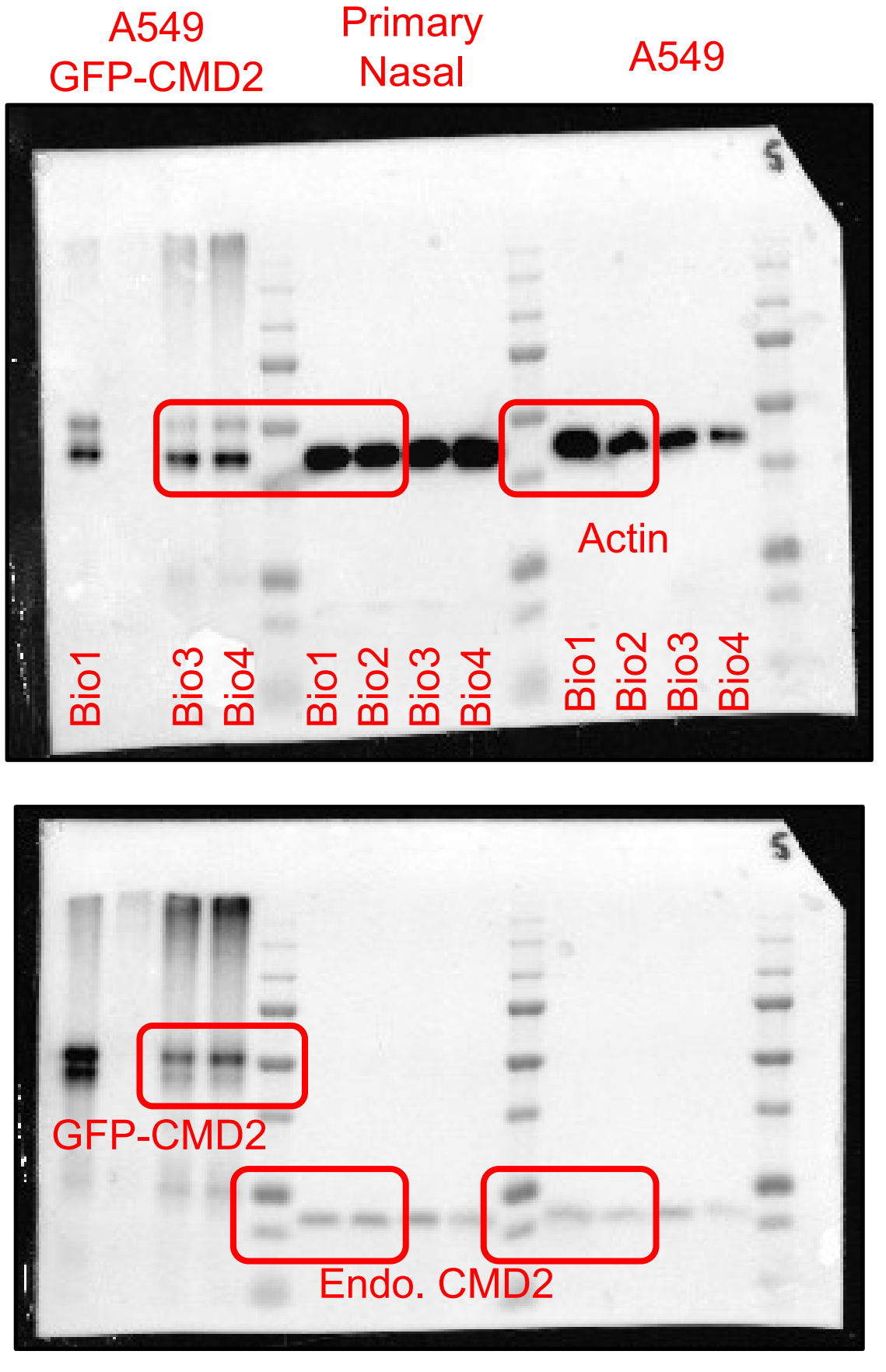

A549
GFP-CMD2
Primary
Nasal
A549
Actin
Bio1
Bio3
Bio4
Bio1
Bio2
Bio3
Bio4
Bio1
Bio2
Bio3
Bio4
GFP-CMD2
Endo. CMD2
