# Supplementary material for: Pneumococcus uses COMMD2 to alter host cellular immunity
Source: Nat Commun. 2026 May 27;17:7733. doi: 10.1038/s41467-026-72852-4 (PMC13434726; doi:10.1038/s41467-026-72852-4)
Supplement: Supplementary file 2 [file 41467_2026_72852_MOESM2_ESM.pdf]

<u>Supplementary Data Table 1: Proteomics data for p65 interactome.</u> All A549 GFP-p65 proteomics data. Sheet 1 contains the color code highlighting proteins of interest (yellow) alongside COMMD proteins, CCDC subunits and Retriever subunits (purple). Sheet 2 has all the statistical comparisons for TIGR4, 6B ST90, and negative (uninfected) GFP-p65 control. Sheet 3 is the quantified phosphosite peptides dataset.

<u>Supplementary Data Table 2: Proteomics data for COMMD2 interactome.</u> All A549 GFP-COMMD2 proteomics data. Sheet 1 contains the color code highlighting proteins of interest (yellow) alongside COMMD proteins, CCDC subunits and Retriever subunits (purple). Sheet 2 has all the statistical comparisons for TIGR4, 6B ST90, IL-1β, uninfected and negative (uninfected) GFP control. Sheet 3 is the quantified ubiquitinated peptides dataset.

<u>Supplementary Data Table 3: Primers and bacterial strains.</u> Data table with all primer information used for cloning and RT-qPCR alongside relevant information for all bacterial strains used in the study.
