# Supplementary material for: Pneumococcus uses COMMD2 to alter host cellular immunity
Source: Nat Commun. 2026 May 27;17:7733. doi: 10.1038/s41467-026-72852-4 (PMC13434726; doi:10.1038/s41467-026-72852-4)
Supplement: Supplementary file 3 [file 41467_2026_72852_MOESM6_ESM.pdf]

nature portfolio

Corresponding author(s): Drs. Michael Connor and Melanie Hamon

Last updated by author(s): Mar 13, 2026

Reporting Summary

Nature Portfolio wishes to improve the reproducibility of the work that we publish. This form provides structure for consistency and transparency in reporting. For further information on Nature Portfolio policies, see our Editorial Policies and the Editorial Policy Checklist.

Statistics

For all statistical analyses, confirm that the following items are present in the figure legend, table legend, main text, or Methods section.

n/a	Confirmed	
☐	☒	The exact sample size (n) for each experimental group/condition, given as a discrete number and unit of measurement
☐	☒	A statement on whether measurements were taken from distinct samples or whether the same sample was measured repeatedly
☐	☒	The statistical test(s) used AND whether they are one- or two-sided<br>*Only common tests should be described solely by name; describe more complex techniques in the Methods section.*
☒	☐	A description of all covariates tested
☐	☒	A description of any assumptions or corrections, such as tests of normality and adjustment for multiple comparisons
☐	☒	A full description of the statistical parameters including central tendency (e.g. means) or other basic estimates (e.g. regression coefficient) AND variation (e.g. standard deviation) or associated estimates of uncertainty (e.g. confidence intervals)
☐	☒	For null hypothesis testing, the test statistic (e.g. F, t, r) with confidence intervals, effect sizes, degrees of freedom and P value noted<br>*Give P values as exact values whenever suitable.*
☒	☐	For Bayesian analysis, information on the choice of priors and Markov chain Monte Carlo settings
☒	☐	For hierarchical and complex designs, identification of the appropriate level for tests and full reporting of outcomes
☒	☐	Estimates of effect sizes (e.g. Cohen's d, Pearson's r), indicating how they were calculated

Our web collection on statistics for biologists contains articles on many of the points above.

Software and code

Policy information about availability of computer code

Data collection	Gen5 BioTek, BioRad Image Lab, MaxQuant (version 1.5.3.8). Software was used generically.
Data analysis	Image Lab, GraphPad Prism 10, GPower 3.1, R studio, FIJI, TAC 4.0 and Microsoft Excel 365 No custom code was used in the manuscript.

For manuscripts utilizing custom algorithms or software that are central to the research but not yet described in published literature, software must be made available to editors and reviewers. We strongly encourage code deposition in a community repository (e.g. GitHub). See the Nature Portfolio guidelines for submitting code & software for further information.

Data

Policy information about availability of data

All manuscripts must include a data availability statement. This statement should provide the following information, where applicable:
- Accession codes, unique identifiers, or web links for publicly available datasets
- A description of any restrictions on data availability
- For clinical datasets or third party data, please ensure that the statement adheres to our policy

The mass spectrometry proteomics data of GFP-p65 and GFP-COMMD2 have been deposited to the ProteomeXchange Consortium via the PRIDE partner repository with the dataset identifiers PXD032970 (p65) and PXD043886 (COMMD2). All data generated in this study are provided in the Supplementary Information/Source Data file.

Research involving human participants, their data, or biological material

Policy information about studies with human participants or human data. See also policy information about sex, gender (identity/presentation), and sexual orientation and race, ethnicity and racism.

Reporting on sex and gender	*Use the terms sex (biological attribute) and gender (shaped by social and cultural circumstances) carefully in order to avoid confusing both terms. Indicate if findings apply to only one sex or gender; describe whether sex and gender were considered in study design; whether sex and/or gender was determined based on self-reporting or assigned and methods used. Provide in the source data disaggregated sex and gender data, where this information has been collected, and if consent has been obtained for sharing of individual-level data; provide overall numbers in this Reporting Summary. Please state if this information has not been collected. Report sex- and gender-based analyses where performed, justify reasons for lack of sex- and gender-based analysis.*
Reporting on race, ethnicity, or other socially relevant groupings	*Please specify the socially constructed or socially relevant categorization variable(s) used in your manuscript and explain why they were used. Please note that such variables should not be used as proxies for other socially constructed/relevant variables (for example, race or ethnicity should not be used as a proxy for socioeconomic status). Provide clear definitions of the relevant terms used, how they were provided (by the participants/respondents, the researchers, or third parties), and the method(s) used to classify people into the different categories (e.g. self-report, census or administrative data, social media data, etc.) Please provide details about how you controlled for confounding variables in your analyses.*
Population characteristics	*Describe the covariate-relevant population characteristics of the human research participants (e.g. age, genotypic information, past and current diagnosis and treatment categories). If you filled out the behavioural & social sciences study design questions and have nothing to add here, write "See above."*
Recruitment	*Describe how participants were recruited. Outline any potential self-selection bias or other biases that may be present and how these are likely to impact results.*
Ethics oversight	*Identify the organization(s) that approved the study protocol.*

Note that full information on the approval of the study protocol must also be provided in the manuscript.

Field-specific reporting

Please select the one below that is the best fit for your research. If you are not sure, read the appropriate sections before making your selection.

[x] Life sciences [ ] Behavioural & social sciences [ ] Ecological, evolutionary & environmental sciences

For a reference copy of the document with all sections, see nature.com/documents/nr-reporting-summary-flat.pdf

Life sciences study design

All studies must disclose on these points even when the disclosure is negative.

Sample size	In vivo experimental sample size was determined and based on previously established power calculations. For in vitro experiments no predetermined sample size calculations were conducted. For these experiments were repeated 3+ biological times with 2-4 technical repeats per biological.
Data exclusions	No data was excluded.
Replication	All experiments, unless otherwise noted, were biologically repeated 3–5 times
Randomization	Animals were randomized into treatment groups prior to in vivo experiments
Blinding	Microscopy was blinded at analysis by the scientist who completed image acquisition.

Reporting for specific materials, systems and methods

We require information from authors about some types of materials, experimental systems and methods used in many studies. Here, indicate whether each material, system or method listed is relevant to your study. If you are not sure if a list item applies to your research, read the appropriate section before selecting a response.

Materials & experimental systems

n/a	Involved in the study
☐	☒ Antibodies
☐	☒ Eukaryotic cell lines
☒	☐ Palaeontology and archaeology
☐	☒ Animals and other organisms
☒	☐ Clinical data
☒	☐ Dual use research of concern
☒	☐ Plants

Methods

n/a	Involved in the study
☒	☐ ChIP-seq
☒	☐ Flow cytometry
☒	☐ MRI-based neuroimaging

Antibodies

Antibodies used	65 (L8F6) (CST ref #6956), p65 phosphorylation at serine 536 (CST ref# 3033), actin AC-15 monoclonal (Sigma ref# A5441), GFP (abcam ref# ab290), GapDH (abcam ref# ab8245), H4 (abcam ref# ab177840), p65 phosphorylation at serine 276 (abcam ref# ab183559), SQSTM1 / p62 antibody [EPR4844] (abcam ref# ab109012), COMMD2 (Sigma ref# HPA044190)
Validation	All antibodies have previously been validated by the source providers with citations, and data present on the manufacturer's website.

Eukaryotic cell lines

Policy information about cell lines and Sex and Gender in Research

Cell line source(s)	A549 cell line was purchased form ATCC. Primary human nasal cells were purchased from PromoCell.
Authentication	None of these cell lines were authenticated in the lab. Commercial data is present from the provider.
Mycoplasma contamination	All cell lines were PCR tested for mycoplasma contamination and found to be negative.
Commonly misidentified lines (See ICLAC register)	None present in manuscript.

Animals and other research organisms

Policy information about studies involving animals; ARRIVE guidelines recommended for reporting animal research, and Sex and Gender in Research

Laboratory animals	Wildtype C57BL/6 male 8-9 week old animals were used for studies.
Wild animals	Studies did not involve wild animals.
Reporting on sex	Only male animals used for survival studies.
Field-collected samples	Studies did not involve field-collected samples.
Ethics oversight	All protocols for animal experiments were reviewed and approved by the CETEA (Comité d'Ethique pour l'Expérimentation Animale - Ethics Committee for Animal Experimentation) of the Institut Pasteur under approval number Dap170005 and were performed in accordance with national laws and institutional guidelines for animal care and use.<br><br>Animals were maintained in the BIME-A3 facility on Institut Pasteur campus which is licensed by the French Ministry of Agriculture (A 75-15-01-1 to -9). Central air conditioning maintains an ambient 22 ± 2 °C, hygrometry at ~55% ±10% and with routine air renewal cycles (at least 20 per hour). Fluorescent light is regulated for a 14:10h light:dark cycle. Animal care is weekly, providing wellness checks, bedding renewal, food and water.

Note that full information on the approval of the study protocol must also be provided in the manuscript.

Plants

Seed stocks

Report on the source of all seed stocks or other plant material used. If applicable, state the seed stock centre and catalogue number. If plant specimens were collected from the field, describe the collection location, date and sampling procedures.

Novel plant genotypes

Describe the methods by which all novel plant genotypes were produced. This includes those generated by transgenic approaches, gene editing, chemical/radiation-based mutagenesis and hybridization. For transgenic lines, describe the transformation method, the number of independent lines analyzed and the generation upon which experiments were performed. For gene-edited lines, describe the editor used, the endogenous sequence targeted for editing, the targeting guide RNA sequence (if applicable) and how the editor was applied.

Authentication

Describe any authentication procedures for each seed stock used or novel genotype generated. Describe any experiments used to assess the effect of a mutation and, where applicable, how potential secondary effects (e.g. second site T-DNA insertions, mosiacism, off-target gene editing) were examined.
