# Supplementary material for: Pneumococcus uses COMMD2 to alter host cellular immunity
Source: Nat Commun. 2026 May 27;17:7733. doi: 10.1038/s41467-026-72852-4 (PMC13434726; doi:10.1038/s41467-026-72852-4)
Supplement: Supplementary file 4 [file 41467_2026_72852_MOESM7_ESM.pdf]

nature portfolio

Peer Review File

Pneumococcus uses COMMD2 to alter host cellular immunity

Corresponding Author: Dr Mélanie Hamon

This file contains all reviewer reports in order by version, followed by all author rebuttals in order by version.

Version 0:

Reviewer comments:

Reviewer #1

(Remarks to the Author)
This study tries to elucidate the molecular mechanisms responsible for the toxicity mediated by specific S. pneumoniae isolates. Salient observations include the demonstration of p65/RelA degradation induced by pathogenic strains, which correlates with blunted NF-kB activation. The model that emerges is one where COMMD2, a member of the COMMD family, is implicated as a mediator of p65/RelA degradation through aggrephagy. Through this mechanism, the authors propose that pathogenic strains cause differential regulation of the NF-kB pathway, attenuating innate immune responses. While interesting on many levels, the paper suffers greatly from incomplete controls in many experiments, which lead to less than optimal technical quality of many experiments. This lack of rigor greatly reduced my confidence in the results presented, and in my opinion, require that many experiments be repeated with proper controls.

Specific comments (in order of data presentation in the paper):
1) To estimate how each condition is affecting the process of RelA phosphorylation, after accounting for equal lane loading (actin), the quantification plots should also include a graph of phosphoRelA/totaRelA. Visually, when accounting for RelA degradation observed in Fig1, the fraction of RelA that is phosphorylated may be similar across all conditions. Thus, the paper should show both p-RelA/actin, and pRelA/RelA after also normalizing for actin.
2) RelA phosphorylation is only one marker of activation of the pathway: RelA acetylation, its binding to promoter regions and other aspects of pathway regulation determine “p65 activation” (line 109). Please be specific in your statement (p65 phosphorylation) and not generic, as other parameters were not assessed.
3) Graphs without standard errors or SD, and without statistical treatment are not interpretable. Fig S1A needs to include at least 3 replicates per time point and condition and should be statistically analyzed. A similar approach should be used for Fig S1B. For readability, I suggest labeling each panel with the cell line model used in each experiment as was done in Fig S1C.
4) Immunofluorescence staining for RelA localization is best suited to detect distribution changes. Quantification of nuclear RelA by IF alone is not reliable and as acknowledged in the paper, is subject to artifacts when nuclear size is also affected. Rather, for quantification purposes, the authors should supplement the analysis with at least 2 additional measures: (1) nuclear fractionation followed by WB analysis for the proteins of interest as was done in Fig 4H when assessing COMMD2. The same should be done for Fig 1E ad F, which should be supplemented with WB for RelA, p50, and potentially all 5 NF-kB subunits), and, (2) kB-site DNA binding capacity in nuclear extracts. The latter is classical physiologic measure of “active” nuclear NF-kB and can be done in many ways, including traditional EMSA with nuclear extracts or DNA binding assays using IP methods with double stranded oligo probes.
5) The data in Figure 2 is fundamentally at odds with the proposed model of a blunted NF-kB response by pathogenic S. pneumoniae isolates.
a. First, Figure 2A mainly shows delayed kinetics of activation and not overall blunting/inhibition of NF-kB activation. Responses to IL-1beta, when cells are exposed to TIGR4, are the same as non-exposed cells at 2 hours. This is confirmed by the ChIP assays at 2 hours shown in Fig 2B, where p65 IP recovered comparable amount of kB-containing promoter regions at 3 pre-specified loci. Thus, the statement in lines 149-151 that TIGR4 antagonizes p65 activation is debatable.
b. I would suggest that the figure should compare target gene induction between pathogenic strains (TIGR4) and non-pathogenic isolates (6B ST90) to substantiate the claim that pathogenicity is associated with differential regulation of NF-kB activation.
6) The use of one pathogenic isolate and its comparison to one non-pathogenic strain is a very limited sample to draw general conclusions on the role of the observed changes in NF-kB activation and COMMD2 participation in the overall mechanisms of S. pneumoniae pathology. Key observations in the paper should be confirmed with a larger set of clinical isolates (3-5 per group) to assess whether this is generalizable to S. pneumoniae or if this is a unique phenomenon of the 2

strains used.
7) The methods for proteomics should be clarified. I would recommend a cartoon depiction of the approach preceding Fig 3A and Fig 7, which would improve readability of the paper. Did the experiments involve biological triplicates or quadruplicates (the methods section is unclear)? Sup table 1 has a list of hits with -Log(P value) and “difference” (presumably Log2 fold change), but it is not clear if this is the TIGR4 exposed cells or the 6B ST90 exposed cells. Both results are discussed in the paper and therefore, both should be shown in this table.
8) Figure 3B seems to try to confirm the proteomic results of Figure 3A showing increased binding of GFP-RelA to RelB in cells exposed to 6B ST90. However, the panel in 3B shows total lysates by WB. Rather, the confirmatory experiment should be a RelA IP followed by RelB WB. This is shown in Fig S2C but the results are not consistent with 3A or 3B: first, the input level of RelB is very different (nearly absent in TIGR4 and 6B ST90 exposed cells) and second, the IP results are misleading as the amount of RelA recovery is not taken into account. To demonstrate differential binding, the recovered RelB should be quantified against IP’d RelA in each condition.
9) Inhibitor experiments used to try to identify the pathways responsible for RelA , COMMD2 and p62 degradation should always include untreated control conditions plus a positive marker protein that is regulated by the inhibited pathway should be shown (eg, a protein sensitive to ubiquitination should be shown when using MG-132 as a control for the effect of the drug during these specific experiments).
10) Recent structural data indicate that COMMD proteins form a ring-like structure (Healy et al, Cell, 2023, and 2 other papers in preprint form with similar conclusions). In these studies, COMMD2 is shown to directly heterodimerize with COMMD3 and to be incorporated in the 10-protein ring. The presence of COMMD4 in the proteomic result suggests that the entire CCC complex is potentially involved. Because of this comment, additional characterization of the interaction induced by TIGR4 is needed: cells should be treated with TIGR4 or the non-pathogenic control line, and P65 should be immunoprecipitated followed by western blots for CCC components, including not only COMMD2 but other COMMD proteins, CCDC22, and CCDC93.
11) The GFP-COMMD2 stable cell line may be expressing supraphysiologic levels of the protein. Thus, a WB for COMMD2 is required to validate the validity of this cell line for the subsequent studies in the paper.
12) The regulation of p62 and COMMD2 localization is interesting (Fig 4C and D). Higher mag images in all 4 conditions should be included.
13) To my knowledge, refs 12 and 22 do not pertain to COMMD1 mediated NF-kB nuclear export. The main nuclear effect of COMMD1 is to promote Cul2-dependent degradation of phosphorylated nuclear RelA (refs 16, 20 in the paper). Rather, a pathway for nucleocytoplasmic trafficking of COMMD1 has been previously demonstrated and this should be cited in relationship to the effects of leptomycin B on COMMD2 localization (PMID: 19220812).
14) I would advise against making any claims on results that are not statistically significant, like the comments about SpxB made in lines 306 to 309 – these should be changed to simply state that the effects were not significant.
15) COMMD2 knockdown level is not acceptable to draw meaningful conclusions. Moreover, any experiment using this technology should include 2 independent oligos and/or should be validated by CRISPR mediated deletion. Similarly, the differences in IL6 and IL8 production shown in Fig 8 are quite small and concerning as far as making robust conclusions. The entire figure thus suffers from many problems and should be repeated when strong knockdown of COMMD2 can be achieved.
16) Proteomic experiments with COMMD2 are confusing. Every proteomic result involving a COMMD protein as a bait results in robust recovery of the CCC and Retriever subunits. At least at baseline, one would have expected this result. This would need to be addressed.
17) RelB and NFKB1 are themselves NF-kB targets: changes in protein abundance may simply reflect transcriptional activity of NF-kB. These results would require RNA analysis by qRT-PCR to understand if the changes observed are post-transcriptional and related to degradation pathways as claimed.
18) Endosomal sorting pathways, which have emerged as a key activity of COMMD proteins and the CCC complex, should received some attention in the discussion. Altered VPS34-dependent PI3P homeostasis is a prominent effect of CCC deficiency (Singla, Nat Comm 2019). This is the same lipid kinase involved in autophagy, and therefore, aggrephagy may be modulated by similar events downstream of COMMD2.
19) The PTMs for p65 should either be shown and validated or should not be part of the paper. At present, the manuscript tries to advance a model for PTM-dependent aggrephagy in the discussion section without showing any data, which would be misleading.

Reviewer #2

(Remarks to the Author)
This is an interesting manuscript proposing a novel NF-kB regulatory pathway that is modulated in cells infected with the virulent S. pneumonia strain, TIGR4. The premise of the paper is that infection with TIGR4 causes aggrephagy-mediated degradation of RelA via COMMD2 and p62, thus suppressing NF-kB pathway activation. This is of interest to the field but some of the experiments outlined are not performed to the standard expected and in many areas, more evidence is needed to support the conclusions drawn.
Examples:

1. In lines 149-151 it is concluded that “the TIGR4 pneumococcal strain antagonizes p65 activation even in the presence of the pro-inflammatory cytokine, IL-1β. This creates a dysfunctional p65 signaling cascade leading to poor downstream activation of p65 dependent transcription”. The conclusion is based on figures 1 and 2 where there is inconsistency between TIGR4 infection +/- IL-1 in western blots and immunocytochemistry. That is, p65 is lost in immunoblots and shows intense nuclear staining by IF. qPCR shows no affect of TIGR4 infection alone on transcription of NF-kB targets (even though there is a significant reduction in total p65 protein) and some reduction in the presence of IL-1 but there are no statistics performed

on these data (Fig 2A). The CHIP qPCR suggests TIGR4 suppresses IL-1 mediated p65 binding at some promoters, but not all. TIGR4 alone has no effect.
The authors should perform more detailed analysis to confirm what TIGR4 infection does to cellular levels and localization of P65 and present a cohesive set of data. Does infection cause degradation of IkB? Reporter assays could be used to determine overall, the effects of TIGR4 infection on NF-kB transcription.

2. Line 187-188: it is concluded “These data, along with the identification of HDAC6 and p62 in the p65 interactome strongly suggest that TIGR4 is inducing degradation of p65 through an aggrephagy pathway”. I was unable to find p62 in supplemental table 1 showing the p65 interactome after TIGR4 infection. The mass spec data for P62 and HDAC6 (FC and p value) should be presented in the figure. Rather than confirm the interaction with HDAC6, the authors perform a number of inhibitor experiments but do not have a DMSO control for each condition, so it is very difficult to draw firm conclusions from these studies. These experiments should be repeated with controls. They should also perform Immunoprecipitation assays to confirm the interaction between p65, HDAC6 and P62
3. Line 254-256 “Therefore, the COMMD2-p65-p62 complex is being degraded through aggrephagy-mediated turnover upon TIGR4 challenge and is amplified in the presence of the pro-inflammatory stimulus IL-1β”. The authors show, using GFP-COMMD2 pull downs, that p62 and p65 bind independently to COMMD2. IF also indicates GFP-COMMD2 and p62 colocalise in nuclear foci after TIGR4 infection. However, there is no evidence of P65 in these foci. Therefore, I am not convinced this conclusion can be drawn based on these data. The immunomicrographs the data in Supplemental figures 3B-D is based on should be shown. If the three proteins formed a complex, you would expect them to appear in aggregates after bafilomycin treatment. Again, DMSO controls should be included. The data in the immunoblots of the IPs in figure 4, with regard to input p65 levels, appear to contradict those in figure 1?
4. Line 270-272 “These surprising data suggest that under nuclear export blockage, which occurs with Leptomycin B treatment , COMMD2 and p62 are naturally recruited to the nucleus at specific puncta through a defined process”. P62, P65 and COMMD proteins are known to shuttle constantly between the nucleus and cytoplasm. Therefore, it is more likely these protein are trapped in the nucleus after lemptomycin treatment. It was obvious p62 and COMMD2 form aggregates but not p65.

Minor
The formatting of graphs is variable throughout and the labels of immunoblots are misaligned in many figures.

There is some very interesting data in the paper and some of the latter data does support the earlier conclusions. I would suggest the authors re-structure the manuscript so that the data that supports their main conclusions is pulled together.

Reviewer #3

(Remarks to the Author)
General comments
Epithelial cells represent the first line of defense against pathogens. The innate immune response initiated by PAMPs sensing PRR initiate a cascade leading mostly to activation of a signal cascade resulting typically in NF-κB activation. Thus, NF-κB is phosphorylated, released from its inhibitory proteins and translocated into the nucleus, thereby inducing transcription of genes encoding cytokines. Cytokines are modulators of the immune response and essential for a balanced host response to control infections.
The presented study shows that pathogenic Streptococcus pneumoniae (serotype TIGR4 pneumococci), which are able to cause invasive infections under in vivo conditions, showed a low NF-κB signaling compared to a pneumococcus (serotype 6B strain; ST90) that is able to colonize the nasopharynx symptomless. TIGR4 leads to degradation of p65 in A549 epithelial cells when infected for 2 hrs, even when A549 were stimulated with IL-1beta. Initial data showed a poor activation of p65 without phosphorylation of S536 and only a low level of S276 phosphorylation. In contrast, both serine residues were phosphorylated in the control experiment with IL-1beta and serotype 6B strain. ChIP-qPCR data suggested that p65 is absent from chromatin under TIGR4 infections. Sophisticated interactome studies revealed a so-called aggrephagy mechanisms involving COMMD2 and p62, a classical receptor for autophagy. Aggrephagy was confirmed by decreased HDAC6 levels during TIGR4 infection of A549 cells but not 6B infections. COMMD2 is required for TIGR4 mediated repression of cytokine production. In contrast, the interactome study with the commensal serotype 6B showed that the sole NF-κB associated target was RelB, part of the non-canonical NF-κB pathway.
COMMD2 with a GFP-tag was immunoprecipitated and p65 and p62 were shown to interact with COMMD2 during TIGR4 infections but not 6B infections. Hence, a complex is formed between COMMD2 and p65. Immunofluorescence imaging and immunoblotting were used to show that COMMD2 was translocated into the nucleus of cells infected with TIGR4. Finally, the study shows that COMMD2 exports p65 from the nucleus to lysosomal degradation, which could be blocked by treatment with Leptomycin B (blocks nuclear export).
The authors also addressed the question which bacterial factors contribute to the TIGR4 effect and used a set of isogenic mutants in their infection model with A549 cells.
TIGR4 strains lacking the capsule showed a similar effect compared to the wild-type, and another 6B strains, which differs in the sequence type (ST138), induced a different COMMD2 translocation compared to 6B ST 90. Hence, the observed effect are serotype-independent.
More importantly, the effect of virulence determinants such as pneumolysin, a pore-forming toxin, and pyruvate oxidase SpxB, generating hydrogen peroxide, was analysed with TIGR4. Data show that pneumolysin contributes significantly toTIGR4 mediated COMMD2 nuclear translocation. SpxB had a minor effect in 2 hrs infection of A549 cells.
Finally, the COMMD2 interactome studies revealed a putative posttranslational modification of COMMD2 knock-down of COMMD2 indeed showed increased levels of IL-8 but not IL-6 suggesting that COMMD2 is involved in gene transcription

General statement:
This is a comprehensive, well written and technically sound study. The results explain for the first time how TIGR4 manipulates the host response and can cause therefore severe invasive diseases while a pneumococcal strain with only commensal potential cannot degrade NF-κB and faces a higher proinflammatory host response compared to TIGR4.
The methods and approaches are clearly described and most of the data are absolutely convincing. However, there are still a few flaws in the presented data, technical questions and overinterpretation of some results.

Specific comments:

1. While the function of COMMD1 and COMMD2 is nicely described, the mechanism of aggrephagy could be introduced (in the results section or earlier) or discussed in more detail, because this is less well-known than autophagy
2. Several of the presented immunoblots are provided with a low resolution. This has to be corrected
3. While convincingly showing that the induced immune response is strikingly different between serotype 6B and TIGR4, it still is not clear, whether adherence of TIGR4 with A549 cells triggers the host response, secreted products are needed or even uptake of TIGR4 contributes to NF-κB degradation
4. The labeling of some Figures like Fig S2C, Fig 4 H, Fig S3 A, is not clear to the reviewer
5. The reviewer is neither an expert in NF-κB signaling nor on COMMD proteins. However, it seems to be that the total level of COMMD2 is even under infectious conditions is similar in all A549 cells (Fig. 4A). However, Fig. 4F shows differences between Un, Il-1beta and TIGR4 infections. Please explain the discrepancies. Is it because the sum of nuclear and cytoplasmic COMMD2 is equal under all conditions?
6. The immunofluorescence microscopy of p62 is convincing, however, the images and differences for COMMD2 under the various conditions are not easy to follow. This is in particular only a few cells are shown, while the graphs (e.g. Fig 5C) show the quantitative data
7. Considering Fig. 6B there is no significant difference between TIGR4 and isogenic capsule knockout
8. Data in Fig. 6C show that pneumolysin contributes to the mechanism. However, all pneumococcal strains produce pneumolysin and serotype 6B will do this as well. This raises the question how TIGR4 pneumolysin influences the NF-κB pathway but not strain 6B? Has the amount or concentration of pneumolysin been measured that is present 2 hrs post infection of A549 cells?
9. Is there a threshold of pneumolysin required to observe the effect?
10. The same is true for SpxB and the production of hydrogen peroxide; or does the data mean there is growth of TIGR4 but not serotype 6B in the infection medium?
11. Can the effect of pneumolysin under the selected conditions been inhibited e.g. by cholesterol or specific antibodies?

Version 1:

Reviewer comments:

Reviewer #1

(Remarks to the Author)
The manuscript is much improved. A few issues remain, which need to be resolved. They are not critical to the key conclusions of the paper so I believe that this should be addressable prior to publication:
- The images for nulear localization of phospho-RelA-S536 in untreated cells are confusing, as they seem to indicate contitutive nuclear localization at high levels which do not match the graph shown. Please review and amend or explain.
- The WB blot to demonstrate the level of expression of GFP-COMMD2 vs the endogenous protein should be shown in the paper and should contain in the same membrane samples from the parental line and the GFP-COMMD2 line. Showing two separate membranes in one panel does not address whether the line expresses physiologic levels of COMMD2.
- In Fig S6, there is a big difference in the mobility of COMMD2 between input and IP lanes which suggests that the band in the IP is not specific. This experiment is problematic as presented.
- The siRNA experiments knocking down COMMD2 produce very modest to minimal knowckdown and therefore are technically problematic.

Open Access This Peer Review File is licensed under a Creative Commons Attribution 4.0 International License, which permits use, sharing, adaptation, distribution and reproduction in any medium or format, as long as you give appropriate credit to the original author(s) and the source, provide a link to the Creative Commons license, and indicate if changes were made.
In cases where reviewers are anonymous, credit should be given to 'Anonymous Referee' and the source.
The images or other third party material in this Peer Review File are included in the article's Creative Commons license, unless indicated otherwise in a credit line to the material. If material is not included in the article's Creative Commons license and your intended use is not permitted by statutory regulation or exceeds the permitted use, you will need to obtain permission directly from the copyright holder.
To view a copy of this license, visit https://creativecommons.org/licenses/by/4.0/

REVIEWER COMMENTS

Reviewer #1 (Remarks to the Author):

This study tries to elucidate the molecular mechanisms responsible for the toxicity mediated by specific S. pneumoniae isolates. Salient observations include the demonstration of p65/RelA degradation induced by pathogenic strains, which correlates with blunted NF-kB activation. The model that emerges is one where COMMD2, a member of the COMMD family, is implicated as a mediator of p65/RelA degradation through aggrephagy. Through this mechanism, the authors propose that pathogenic strains cause differential regulation of the NF-kB pathway, attenuating innate immune responses. While interesting on many levels, the paper suffers greatly from incomplete controls in many experiments, which lead to less than optimal technical quality of many experiments. This lack of rigor greatly reduced my confidence in the results presented, and in my opinion, require that many experiments be repeated with proper controls.
We appreciate the very detailed and constructive comments by the reviewer regarding NF-kB signaling. Our main objective in this manuscript was to focus on the role of COMMD2 on pneumococcal virulence, and not necessarily NF-kB engagement directly. We realize from this reviewer's comments that this did not come through in our last manuscript. Therefore, along with incorporating most of the reviewer's comments, we have revised the structure of our manuscript, placing more emphasis on COMMD2.

Specific comments (in order of data presentation in the paper):
1) To estimate how each condition is affecting the process of RelA phosphorylation, after accounting for equal lane loading (actin), the quantification plots should also include a graph of phosphoRelA/totaRelA. Visually, when accounting for RelA degradation observed in Fig1, the fraction of RelA that is phosphorylated may be similar across all conditions. Thus, the paper should show both p-RelA/actin, and pRelA/RelA after also normalizing for actin.
Thank you for this suggestion. We have implemented quantifications in the manuscript to show the ratios in figure 2.

2) RelA phosphorylation is only one marker of activation of the pathway: RelA acetylation, its binding to promoter regions and other aspects of pathway regulation determine "p65 activation" (line 109). Please be specific in your statement (p65 phosphorylation) and not generic, as other parameters were not assessed.
We have corrected this statement to reflect only phosphorylation.

3) Graphs without standard errors or SD, and without statistical treatment are not interpretable. Fig S1A needs to include at least 3 replicates per time point and condition and should be statistically analyzed. A similar approach should be used for Fig S1B. For readability, I suggest labeling each panel with the cell line model used in each experiment as was done in Fig S1C.
This comment no longer applies to the new manuscript structure as the original S1A figure was replaced with time course microscopy (Fig 2) in the new manuscript. We have incorporated the suggestion to label the cell line used.

4) Immunofluorescence staining for RelA localization is best suited to detect distribution changes. Quantification of nuclear RelA by IF alone is not reliable and as acknowledged in the paper, is subject to artifacts when nuclear size is also affected. Rather, for quantification purposes, the authors should supplement the analysis with at least 2 additional measures: (1) nuclear fractionation followed by WB analysis for the proteins of interest as was done in Fig 4H when assessing COMMD2. The same should be done for Fig 1E ad F, which should be supplemented with WB for RelA, p50, and potentially all 5 NF-kB subunits), and, (2) kB-site DNA binding capacity in nuclear extracts. The latter is classical physiologic measure of "active" nuclear NF-kB and can be done in many ways, including traditional EMSA with nuclear extracts or DNA binding assays using IP methods with double stranded oligo probes.
We agree with the reviewer that our characterization of NF-kB activation was not very detailed. Indeed, we wanted to focus mainly on the role of COMMD2 and not on NF-kB, which is why we have now changed the structure of the manuscript. However, while this comment no longer directly applies to the new manuscript structure, we have incorporated mean intensity measurements as opposed to integrated intensity (intensity over area) when the focus of statistical comparison of pneumococcal challenge is to unchallenged cells. These data further argue that when comparing pneumococcal challenge groups across each other integrated intensity (intensity of area) is acceptable. Below is data showing the impact of infection on nuclear size and nuclear intensity measurements.

The results show TIGR4 does significantly reduce nuclear size in comparison to uninfected, thus when comparing TIGR4 to mock-like conditions the mean intensity should be used. However cross-comparisons of pneumococcal challenge conditions integrated intensity is sufficient. These data are provided for the reviewer but are not included within the revised manuscript.

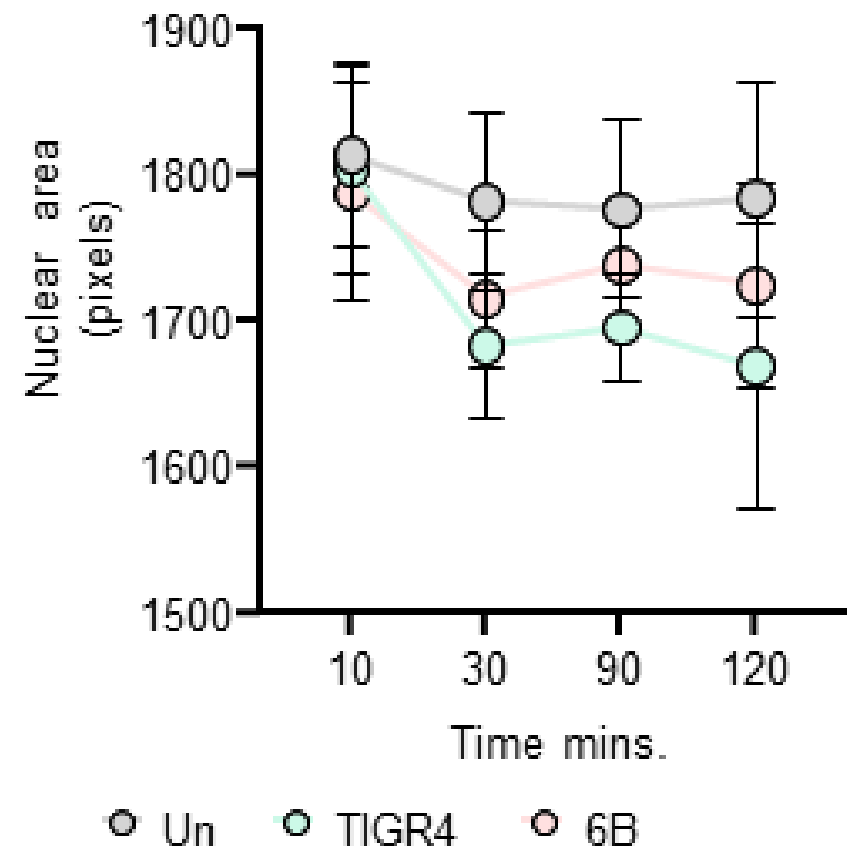

T.Test to uninfected

Area	10	30	90	120
TIGR4	0.752	0.008	0.001	0.001
6B ST90	0.759	0.084	0.095	0.197

5) The data in Figure 2 is fundamentally at odds with the proposed model of a blunted NF-kB response by pathogenic S. pneumoniae isolates.

a. First, Figure 2A mainly shows delayed kinetics of activation and not overall blunting/inhibition of NF-kB activation. Responses to IL-1beta, when cells are exposed to TIGR4, are the same as non-exposed cells at 2 hours. This is confirmed by the ChIP assays at 2 hours shown in Fig 2B, where p65 IP recovered comparable amount of kB-containing promoter regions at 3 pre-specified loci. Thus, the statement in lines 149-151 that TIGR4 antagonizes p65 activation is debatable.

We agree with the reviewer that our statement was too strong, and in fact is not the main focus of our manuscript. We have re-worded our statements on this matter in the new manuscript to take this point into account.

b. I would suggest that the figure should compare target gene induction between pathogenic strains (TIGR4) and non-pathogenic isolates (6B ST90) to substantiate the claim that pathogenicity is associated with differential regulation of NF-kB activation.

While this comment no longer directly applies to the new manuscript structure, we have incorporated additional kinetic experiments for p65 phos S536 (Fig. 2). We also completed S276 kinetics (below), but this is not included within the manuscript. However, the data is comparable to our western blot results at 2hrs located in Fig. 2. Panel D, and Sup. Fig. 2 panel B.

Moreover, we kindly draw the reviewer's attention to our previous publication where we completed a time course experiment of RT-qPCR directly comparing TIGR4 and 6B. We have also updated the manuscript text to directly reflect this previous study. (Connor, M. G. et al. The histone demethylase KDM6B fine-tunes the host response to Streptococcus pneumoniae. Nat Microbiol 6, 257-269, doi:10.1038/s41564-020-00805-8 (2021).)

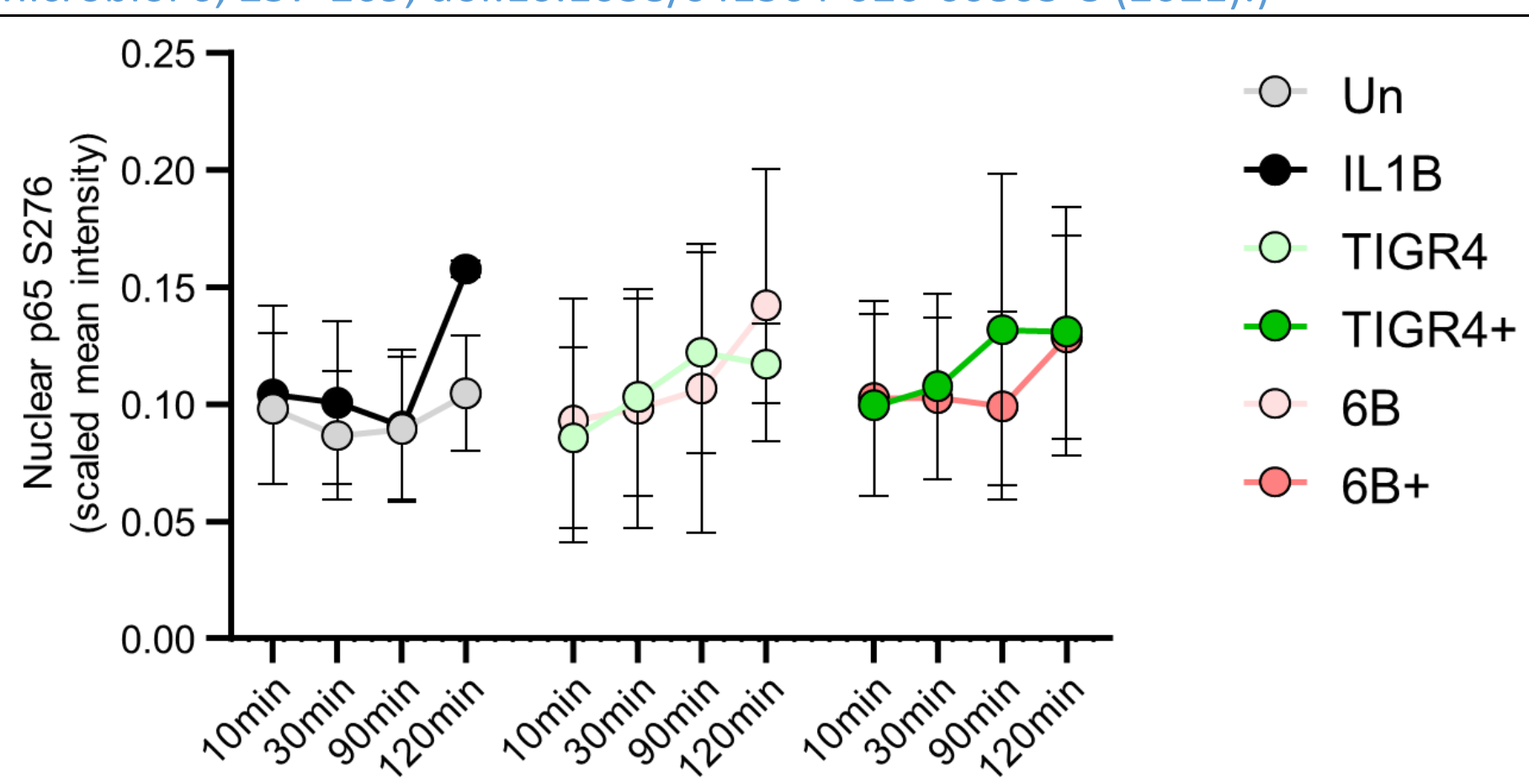

6) The use of one pathogenic isolate and its comparison to one non-pathogenic strain is a very limited sample to draw general conclusions on the role of the observed changes in NF-kB activation and COMMD2 participation in the overall mechanisms of S. pneumoniae pathology. Key observations in the paper should be confirmed with a larger set of clinical isolates (3-5 per group) to assess whether this is generalizable to S. pneumoniae or if this is a unique phenomenon of the 2 strains used.
We appreciate the reviewer's comments and agree. We have added such comparisons in the new manuscript (Fig. 8), which we believe considerably strengthens our conclusions.

7) The methods for proteomics should be clarified. I would recommend a cartoon depiction of the approach preceding Fig 3A and Fig 7, which would improve readability of the paper. Did the experiments involve biological triplicates or quadruplicates (the methods section is unclear)? Sup table 1 has a list of hits with -Log(P value) and "difference" (presumably Log2 fold change), but it is not clear if this is the TIGR4 exposed cells or the 6B ST90 exposed cells. Both results are discussed in the paper and therefore, both should be shown in this table.
We agree and have: 1) reanalyzed both the p65 (Sup. Table 1) and COMMD2 (Sup. Table 2) proteomics data in the same manner, 2) provided experimental diagrams for each study, 3) displayed all volcano plots related to comparison, and 4) updated the supplemental tables to include 2 sheets; one with all the data and a second with PTMs mentioned in the discussion. Pointedly, these two mass spectrometry experiments were completed by two independent groups, which attests to the strength of the datasets when they are cross compared.

8) Figure 3B seems to try to confirm the proteomic results of Figure 3A showing increased binding of GFP-RelA to RelB in cells exposed to 6B ST90. However, the panel in 3B shows total lysates by WB. Rather, the confirmatory experiment should be a RelA IP followed by RelB WB. This is shown in Fig S2C but the results are not consistent with 3A or 3B: first, the input level of RelB is very different (nearly absent in TIGR4 and 6B ST90 exposed cells) and second, the IP results are misleading as the amount of RelA recovery is not taken into account. To demonstrate differential binding, the recovered RelB should be quantified against IP'd RelA in each condition.
Within the new manuscript structure, we believe including RelB data was no longer important for our conclusions and was removed.

9) Inhibitor experiments used to try to identify the pathways responsible for RelA , COMMD2 and p62 degradation should always include untreated control conditions plus a positive marker protein that is regulated by the inhibited pathway should be shown (eg, a protein sensitive to ubiquitination should be shown when using MG-132 as a control for the effect of the drug during these specific experiments).
We believe in the new manuscript structure the inclusion of the additional inhibitors was no longer important for our conclusions and were removed.

10) Recent structural data indicate that COMMD proteins form a ring-like structure (Healy et al, Cell, 2023, and 2 other papers in preprint form with similar conclusions). In these studies, COMMD2 is shown to directly heterodimerize with COMMD3 and to be incorporated in the 10-protein ring. The presence of COMMD4 in the proteomic result suggests that the entire CCC complex is potentially involved. Because of this comment, additional characterization of the interaction induced by TIGR4 is needed: cells should be treated with TIGR4 or the non-pathogenic control line, and P65 should be immunoprecipitated followed by western blots for CCC components, including not only COMMD2 but other COMMD proteins, CCDC22, and CCDC93.
In our new manuscript, at the suggestion of the other reviewers and the editor, we focused on the relationship between COMMD2 and pneumococcal virulence, with the others being beyond the scope of this publication. However, in our p65 proteomics data (Sup. Table 1) there is COMMD4, along with VPS35L, CCDC93, COMMD9, COMMD6, VPS29, CCDC22 & COMMD1. Of these, COMMD4, VPS35L & CCDC93 are significantly enriched within the TIGR4 dataset. Moreover, our COMMD2 proteomics data (Sup. Table 2) enriched for: CCDC22, CDC93, VPS35L, COMMD6, COMMD2, COMMD1, COMMD4, COMMD8, COMMD9, COMMD3 & VPS29. Given the overlap between the two proteomic datasets, and our focus, we have not done the additional immunoprecipitation studies to blot for the other COMMD, CCC or Retriever proteins. As mentioned above, these two mass spectrometry experiments were completed by two independent groups. We have high confidence in the identified interacting partners and the subsequent overlap of the datasets.

We have also included the Healy et al, Cell, 2023 citation within the manuscript within the COMMD2 proteomics section (Lines279-283).

11) The GFP-COMMD2 stable cell line may be expressing supraphysiologic levels of the protein. Thus, a WB for COMMD2 is required to validate the validity of this cell line for the subsequent studies in the paper.
We appreciate the reviewers' concern of supraphysiologic levels of COMMD2 in our ectopic expression cell line.

Our GFP-COMMD2 cell line was constructed using the highly robust Sleeping Beauty system (ref below), which uses a transposon for genomic integration into the cell chromosome. This method is further known to limit the number of integrates at ~ 10-12. To make our cell line we further used the GFP expression to select cell clones that displayed robust signal against the negative A549 control (between 10^3 to $<10^4$ arbitrary fluorescent units) but were not overwhelming bright by FACS. Due to this method, and our approach to stable cell line generation, conventional concerns of supraphysiological expression are lower, as we are not selecting for the brightest clones in the population.

Nonetheless, we have sampled our uninfected whole cell lysates and probed them for COMMD2 and actin. The data below shows that the GFP-CMD2 cell line comparison to the parental A549 cell line and primary human nasal cells have comparable levels of COMMD2. While a statistically significant difference is reported between the A549 GFP-CMD2 cell line and the parental A549 strain we do not believe it to be biologically influential as it is both a minimal difference and is not significantly different to the primary human nasal cells. Importantly, in the ectopic GFP-COMMD2 expression cell line the amount of endogenous COMMD2 is minimal. These data are provided for the reviewer only and are not in the current manuscript.

Ref:
Kowarz, E., Loscher, D. & Marschalek, R. Optimized Sleeping Beauty transposons rapidly generate stable transgenic cell lines. Biotechnology journal 10, 647-653, doi:10.1002/biot.201400821 (2015).

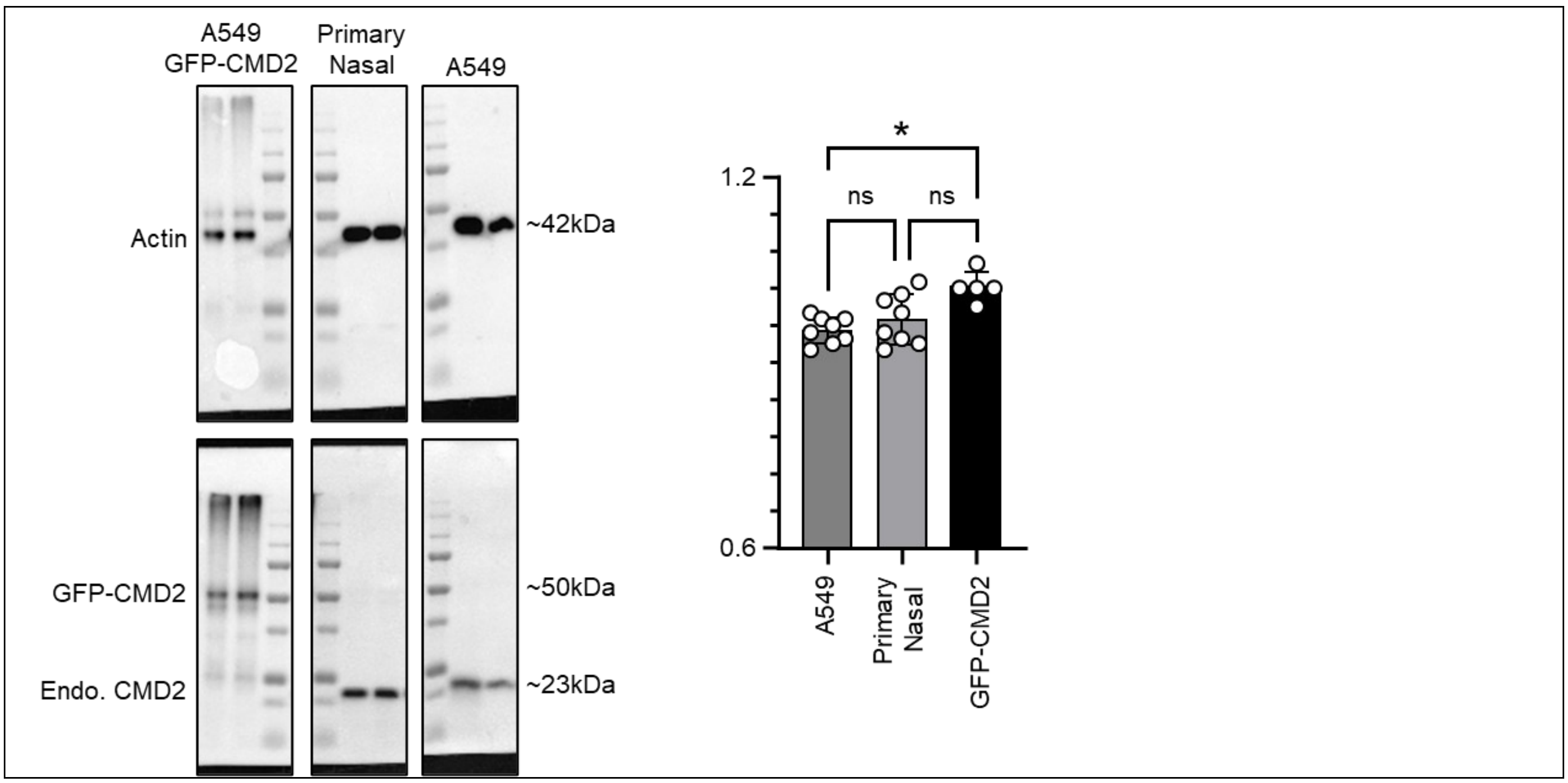

12) The regulation of p62 and COMMD2 localization is interesting (Fig 4C and D). Higher mag images in all 4 conditions should be included.
In the new manuscript structure this original dataset referred to by the reviewer was removed as we believe it did not impact our conclusions considering our new Figure 3. Moreover, the results reported in our immunoprecipitation (Fig.4 & Sup. Fig. 6) and both mass spectrometry datasets (Sup. Tables 1&2), which show interaction of COMMD2, p62

(SQSTM1) and p65. However, we have provided below higher magnification images from the removed dataset from the original manuscript below, with white arrows highlighting overlap for the reviewer.

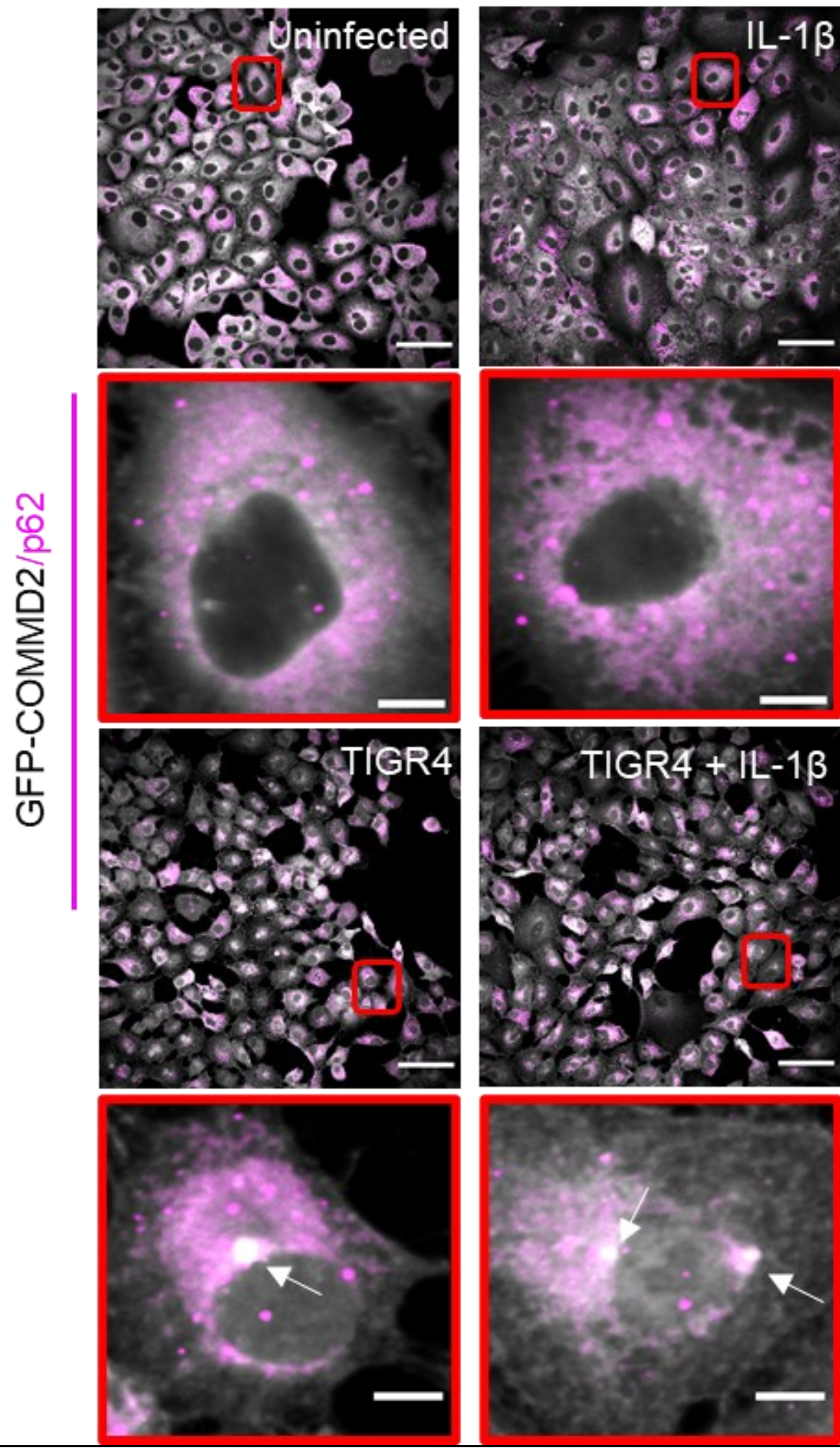

13) To my knowledge, refs 12 and 22 do not pertain to COMMD1 mediated NF-kB nuclear export. The main nuclear effect of COMMD1 is to promote Cul2-dependent degradation of phosphorylated nuclear RelA (refs 16, 20 in the paper). Rather, a pathway for nucleocytoplasmic trafficking of COMMD1 has been previously demonstrated and this should be cited in relationship to the effects of leptomycin B on COMMD2 localization (PMID: 19220812).
We thank the reviewer for this and have now included this in the new manuscript.

14) I would advise against making any claims on results that are not statistically significant, like the comments about SpxB made in lines 306 to 309 – these should be changed to simply state that the effects were not significant.
We have now extensively addressed this comment in the new manuscript structure in Fig 8 and Sup. Fig 10. We show that the synergistic effect of PLY with SpxB is dependent on pore formation. These results show for the first time the Pneumolysin toxin may mask other bacterial protein effectors. This significantly advances the pneumococcal field by providing evidence that pore formation may indeed be a passive "conduit" allowing entry into the host cell of other unknown virulence associated bacterial proteins.

15) COMMD2 knockdown level is not acceptable to draw meaningful conclusions. Moreover, any experiment using this technology should include 2 independent oligos and/or should be validated by CRISPR mediated deletion. Similarly, the differences in IL6 and IL8 production shown in Fig 8 are quite small and concerning as far as making robust conclusions. The entire figure thus suffers from many problems and should be repeated when strong knockdown of COMMD2 can be achieved.
We appreciate the reviewer's concerns and have since used an additional oligo (Sup. Fig. 9). This additional oligo induced ~ 50% knockdown of COMMD2 and was subsequently used for additional experiments. We have also completed Luminex of 27 analytes (Fig. 7; Sup. Fig. 9). We did not perform CRISPR deletion as we believe that it would remove the

encoded protein completely as opposed to RNAi mediated knockdown which only lowers levels in a transient manner. From our previous experiences in pneumococcal infection biology our RNAi approach is more beneficial in assessing impacts host proteins have on the outcomes of host phenotypes during challenge as opposed to complete deletion.

16) Proteomic experiments with COMMD2 are confusing. Every proteomic result involving a COMMD protein as a bait results in robust recovery of the CCC and Retriever subunits. At least at baseline, one would have expected this result. This would need to be addressed.
We have provided a new volcano plot showing GFP-COMMD2 vs. GFP alone (Sup. Fig 8), and updated the COMMD2 (Sup. Table 2) proteomics data. Within the COMMD2 proteomics data set we have highlighted in purple additional COMMD proteins, CCDC subunits and Retriever subunits. These data show: CCDC22, CDC93, VPS35L, COMMD6, COMMD2, COMMD1, COMMD4, COMMD8, COMMD9, COMMD3 & VPS29 are present. Additionally, in our p65 proteomics data (Sup. Table 1) VPS35L, CCDC93, COMMD4, COMMD9, COMMD6, VPS29, CCDC22 & COMMD1 were identified. Altogether, all our mass spec data is in-line with your comment.

17) RelB and NFKB1 are themselves NF-kB targets: changes in protein abundance may simply reflect transcriptional activity of NF-kB. These results would require RNA analysis by qRT-PCR to understand if the changes observed are post-transcriptional and related to degradation pathways as claimed.
As we have restructured the manuscript we feel additional RT-qPCR focused on these targets is no longer needed for our conclusions. However, we have previously established that the pneumococcal strain 6B ST90 upregulates the *RelB* transcript in A549 cells in comparison to the TIGR4 strain. (Connor, M. G. et al. The histone demethylase KDM6B fine-tunes the host response to Streptococcus pneumoniae. Nat Microbiol 6, 257-269, doi:10.1038/s41564-020-00805-8 (2021).)

18) Endosomal sorting pathways, which have emerged as a key activity of COMMD proteins and the CCC complex, should received some attention in the discussion. Altered VPS34-dependent PI3P homeostasis is a prominent effect of CCC deficiency (Singla, Nat Comm 2019). This is the same lipid kinase involved in autophagy, and therefore, aggrephagy may be modulated by similar events downstream of COMMD2.
We are grateful for this point by the reviewer. For transparency, VPS34 is not present in either of our mass spec datasets. However, VPS35L along with several others are present. Nonetheless, we have now included this concept within the context of pneumococcal infection within the discussion in Lines 460-465.

19) The PTMs for p65 should either be shown and validated or should not be part of the paper. At present, the manuscript tries to advance a model for PTM-dependent aggrephagy in the discussion section without showing any data, which would be misleading.
We appreciate the reviewer's concern and have now placed this section in the discussion of the revised manuscript. We believe it is important to discuss this point for our future studies. Although we would have liked to perform in detail analyses of these modifications, the PTMs we focus on S203 and S45 have no commercial antibodies, which makes their study complicated and outside the scope of the current work.

Reviewer #2 (Remarks to the Author):
This is an interesting manuscript proposing a novel NF-kB regulatory pathway that is modulated in cells infected with the virulent S. pneumonia strain, TIGR4. The premise of the paper is that infection with TIGR4 causes aggrephagy-mediated degradation of RelA via COMMD2 and p62, thus suppressing NF-kB pathway activation. This is of interest to the field but some of the experiments outlined are not performed to the standard expected and in many areas, more evidence is needed to support the conclusions drawn.

Examples:
1. In lines 149-151 it is concluded that "the TIGR4 pneumococcal strain antagonizes p65 activation even in the presence of the pro-inflammatory cytokine, IL-1β. This creates a dysfunctional p65 signaling cascade leading to poor downstream activation of p65 dependent transcription". The conclusion is based on figures 1 and 2 where there is inconsistency between TIGR4 infection +/- IL-1 in western blots and immunocytochemistry. That is, p65 is lost in immunoblots and shows intense nuclear staining by IF. qPCR shows no affect of TIGR4 infection alone on transcription of NF-kB targets (even though there is a significant reduction in total p65 protein) and some reduction in the presence of IL-1 but there

are no statistics performed on these data (Fig 2A). The CHIP qPCR suggests TIGR4 suppresses IL-1 mediated p65 binding at some promoters, but not all. TIGR4 alone has no effect.

The authors should perform more detailed analysis to confirm what TIGR4 infection does to cellular levels and localization of P65 and present a cohesive set of data. Does infection cause degradation of IkB? Reporter assays could be used to determine overall, the effects of TIGR4 infection on NF-kB transcription.
In the new manuscript structure, we have provided additional statistics and evidence for our claims across Figures 1 & 2, this includes kinetics of p65 S536 and additional statistics across the qPCR data. However, with this new focus we do not feel following up with IkB is needed to support our claims.

2. Line 187-188: it is concluded "These data, along with the identification of HDAC6 and p62 in the p65 interactome strongly suggest that TIGR4 is inducing degradation of p65 through an aggrephagy pathway". I was unable to find p62 in supplemental table 1 showing the p65 interactome after TIGR4 infection. The mass spec data for P62 and HDAC6 (FC and p value) should be presented in the figure. Rather than confirm the interaction with HDAC6, the authors perform a number of inhibitor experiments but do not have a DMSO control for each condition, so it is very difficult to draw firm conclusions from these studies. These experiments should be repeated with controls. They should also perform Immunoprecipitation assays to confirm the interaction between p65, HDAC6 and P62
We apologize for this and have now addressed the mass spec table. The reviewer will find both HDAC6 and SQSTM1 in the new Sup. Table 1 Mass_spec_p65. Moreover, we had this dataset analyzed to generate new volcano plots and biological function comparisons (Figure 1 and Sup. Figure 1). These data now clearly show HDAC6 and SQSTM1 in comparison to 6B and GFP alongside biological processes analysis enrichment for aggrephagy. Given this, in the new manuscript structure the inclusion of the additional inhibitors was no longer important for our conclusions and were removed.

3. Line 254-256 "Therefore, the COMMD2-p65-p62 complex is being degraded through aggrephagy-mediated turnover upon TIGR4 challenge and is amplified in the presence of the pro-inflammatory stimulus IL-1β". The authors show, using GFP-COMMD2 pull downs, that p62 and p65 bind independently to COMMD2. IF also indicates GFP-COMMD2 and p62 colocalise in nuclear foci after TIGR4 infection. However, there is no evidence of P65 in these foci. Therefore, I am not convinced this conclusion can be drawn based on these data. The immunomicrographs the data in Supplemental figures 3B-D is based on should be shown. If the three proteins formed a complex, you would expect them to appear in aggregates after bafilomycin treatment. Again, DMSO controls should be included. The data in the immunoblots of the IPs in figure 4, with regard to input p65 levels, appear to contradict those in figure 1?
We have now added endogenous IP of p65 showing COMMD2 (Sup. Fig. 6) to demonstrate that the complex is also present during native conditions during TIGR4 challenge. The commercial COMMD2 antibody does not work in IF or IP.

4. Line 270-272 "These surprising data suggest that under nuclear export blockage, which occurs with Leptomycin B treatment, COMMD2 and p62 are naturally recruited to the nucleus at specific puncta through a defined process". P62, P65 and COMMD proteins are known to shuttle constantly between the nucleus and cytoplasm. Therefore, it is more likely these protein are trapped in the nucleus after lemptomycin treatment. It was obvious p62 and COMMD2 form aggregates but not p65.
We have updated our results, now Fig. 5 in the new manuscript, to show tangent line traces of COMMD2 with either p65 or p62 as a measure of co-association. While p65 nuclear – cytoplasmic oscillation occurs, we are the first to show COMMD2 nuclear translocation and this coincides with increase of p62 under these conditions. We acknowledge there are multiple means to export targets from the nucleus. However, we specifically use leptomycin B to block CRM1 export. Thus, targets that require CRM1 for export would result in their accumulation – as we show for COMMD2, p65 and p62. Moreover, the dramatic change in amounts of p65 vs p62 and COMMD2 under Leptomycin B treatment further strengthens that nuclear – cytoplasmic oscillation of p62 and COMMD2 does not occur naturally at a comparable rate as p65.

Minor
The formatting of graphs is variable throughout and the labels of immunoblots are misaligned in many figures.
There is some very interesting data in the paper and some of the latter data does support the earlier conclusions. I would suggest the authors re-structure the manuscript so that the data that supports their main conclusions is pulled

together.
We thank you for this advice, and we have done so in this new manuscript. Please note, if any, of these new figures have alignment issues, and we will gladly correct them.

Reviewer #3 (Remarks to the Author):
General comments
Epithelial cells represent the first line of defense against pathogens. The innate immune response initiated by PAMPs sensing PRR initiate a cascade leading mostly to activation of a signal cascade resulting typically in NF-κB activation. Thus, NF-κB is phosphorylated, released from its inhibitory proteins and translocated into the nucleus, thereby inducing transcription of genes encoding cytokines. Cytokines are modulators of the immune response and essential for a balanced host response to control infections.
The presented study shows that pathogenic Streptococcus pneumoniae (serotype TIGR4 pneumococci), which are able to cause invasive infections under in vivo conditions, showed a low NF-κB signaling compared to a pneumococcus (serotype 6B strain; ST90) that is able to colonize the nasopharynx symptomless. TIGR4 leads to degradation of p65 in A549 epithelial cells when infected for 2 hrs, even when A549 were stimulated with IL-1beta. Initial data showed a poor activation of p65 without phosphorylation of S536 and only a low level of S276 phosphorylation. In contrast, both serine residues were phosphorylated in the control experiment with IL-1beta and serotype 6B strain. ChIP-qPCR data suggested that p65 is absent from chromatin under TIGR4 infections. Sophisticated interactome studies revealed a so-called aggrephagy mechanisms involving COMMD2 and p62, a classical receptor for autophagy. Aggrephagy was confirmed by decreased HDAC6 levels during TIGR4 infection of A549 cells but not 6B infections. COMMD2 is required for TIGR4 mediated repression of cytokine production. In contrast, the interactome study with the commensal serotype 6B showed that the sole NF-κB associated target was RelB, part of the non-canonical NF-κB pathway.
COMMD2 with a GFP-tag was immunoprecipitated and p65 and p62 were shown to interact with COMMD2 during TIGR4 infections but not 6B infections. Hence, a complex is formed between COMMD2 and p65. Immunofluorescence imaging and immunoblotting were used to show that COMMD2 was translocated into the nucleus of cells infected with TIGR4. Finally, the study shows that COMMD2 exports p65 from the nucleus to lysosomal degradation, which could be blocked by treatment with Leptomycin B (blocks nuclear export).
The authors also addressed the question which bacterial factors contribute to the TIGR4 effect and used a set of isogenic mutants in their infection model with A549 cells.
TIGR4 strains lacking the capsule showed a similar effect compared to the wild-type, and another 6B strains, which differs in the sequence type (ST138), induced a different COMMD2 translocation compared to 6B ST 90. Hence, the observed effect are serotype-independent.
More importantly, the effect of virulence determinants such as pneumolysin, a pore-forming toxin, and pyruvate oxidase SpxB, generating hydrogen peroxide, was analysed with TIGR4. Data show that pneumolysin contributes significantly toTIGR4 mediated COMMD2 nuclear translocation. SpxB had a minor effect in 2 hrs infection of A549 cells.
Finally, the COMMD2 interactome studies revealed a putative posttranslational modification of COMMD2 knock-down of COMMD2 indeed showed increased levels of IL-8 but not IL-6 suggesting that COMMD2 is involved in gene transcription

General statement:
This is a comprehensive, well written and technically sound study. The results explain for the first time how TIGR4 manipulates the host response and can cause therefore severe invasive diseases while a pneumococcal strain with only commensal potential cannot degrade NF-κB and faces a higher proinflammatory host response compared to TIGR4. The methods and approaches are clearly described and most of the data are absolutely convincing. However, there are still a few flaws in the presented data, technical questions and overinterpretation of some results.
We appreciate your concise summary of our findings and your concerns. In this completely restructured manuscript, we have included substantial additional data to support our claims and address your concerns.

Specific comments:
1. While the function of COMMD1 and COMMD2 is nicely described, the mechanism of aggrephagy could be introduced (in the results section or earlier) or discussed in more detail, because this is less well-known than autophagy.
Thank you for these suggestions, we have now added the requested information in this new manuscript. For clarity, aggrephagy is a selective form of autophagy (an umbrella term) for degradation of proteins.

2. Several of the presented immunoblots are provided with a low resolution. This has to be corrected

We kindly ask the reviewer to denote which of the immunoblots, if any, in this new manuscript are not of sufficient resolution – we will gladly provide these. All full immunoblots are in the supplemental information as well.

3. While convincingly showing that the induced immune response is strikingly different between serotype 6B and TIGR4, it still is not clear, whether adherence of TIGR4 with A549 cells triggers the host response, secreted products are needed or even uptake of TIGR4 contributes to NF-κB degradation

We appreciate this insightful remark. We have addressed this point in the new manuscript in Fig 8 and Sup. Fig 10. We have now added additional data to provide evidence that the pore formation of PLY is required along with SpxB for the full effect. Furthermore, no differences in recovered CFUs 2hrs post-challenge of A549 cells was observed for TIGR4 vs. 6B (see data graph comment 10). Altogether, these data provide compelling evidence that individually PLY and SpxB are not sufficient, but work in tandem for the effect.

4. The labeling of some Figures like Fig S2C, Fig 4 H, Fig S3 A, is not clear to the reviewer

Thank you for pointing these specific figures out. In the new manuscript, Fig S2C is no longer included. However, for Fig4H and S3A (which are now Sup. Fig 3A and Sup. Fig. 3C) we have clarified the labeling. If any other panes are unclear, please let us know, we will gladly correct these.

5. The reviewer is neither an expert in NF-κB signaling nor on COMMD proteins. However, it seems to be that the total level of COMMD2 is even under infectious conditions is similar in all A549 cells (Fig. 4A). However, Fig. 4F shows differences between Un, Il-1beta and TIGR4 infections. Please explain the discrepancies. Is it because the sum of nuclear and cytoplasmic COMMD2 is equal under all conditions?

We removed the original figure 4F as it did not change our conclusions in the new manuscript structure with Fig.3. To address the reviewer comment on the disparity in total COMMD2 levels – we believe it is a combination of the total sum of cytoplasmic and nuclear alongside turnover at these timepoints. Pointedly, even in our GFP-COMMD2 ectopic expression cell line there is ~ 40% reduction in total GFP-COMMD2 in the IP input samples. This parallels the reduction seen in microscopy after we plotted mean intensity instead of integrated intensity (intensity over area), as TIGR4 challenge significantly reduces the nuclear size which introduces a mathematical bias to the integrated intensity.

6. The immunofluorescence microscopy of p62 is convincing, however, the images and differences for COMMD2 under the various conditions are not easy to follow. This is in particular only a few cells are shown, while the graphs (e.g. Fig 5C) show the quantitative data

We have updated our results for Fig. 5 in the new manuscript, to show tangent line traces of COMMD2 with either p65 or p62 as a measure of co-association alongside new representative images. Given the substantial increase in background in nuclear retention of p65 we sided against conventional co-location (or co-association) scoring via a Mander's of Overlap coefficient as to not misled the reader.

7. Considering Fig. 6B there is no significant difference between TIGR4 and isogenic capsule knockout

We thank the reviewer for this comment. Indeed, that is the case, we have added additional statistical comparisons to the new Fig. 8.

8. Data in Fig. 6C show that pneumolysin contributes to the mechanism. However, all pneumococcal strains produce pneumolysin and serotype 6B will do this as well. This raises the question how TIGR4 pneumolysin influences the NF-κB pathway but not strain 6B? Has the amount or concentration of pneumolysin been measured that is present 2 hrs post infection of A549 cells?

We agree that this point is confusing and in fact, we previously demonstrated that TIGR4 and 6B ST90 have similar levels of PLY (Connor, M. G. et al. The histone demethylase KDM6B fine-tunes the host response to Streptococcus pneumoniae. Nat Microbiol 6, 257-269, doi:10.1038/s41564-020-00805-8 (2021)). We have now added data showing that not only PLY is required but also SpxB. Therefore, other factors are required and could explain the differences between the strains.

9. Is there a threshold of pneumolysin required to observe the effect?

We do not believe there is a threshold of PLY required for the COMMD2 phenotype. We have titrated purified PLY toxin with a TIGR4ΔSpx Δply (n=2 biological; 4 technicals per). The results of these studies clearly show PLY alone does not induce COMMD2 nuclear translocation. Moreover, this is additional evidence that both PLY and SpxB are necessary for the full effect. These data are not included in the new manuscript.

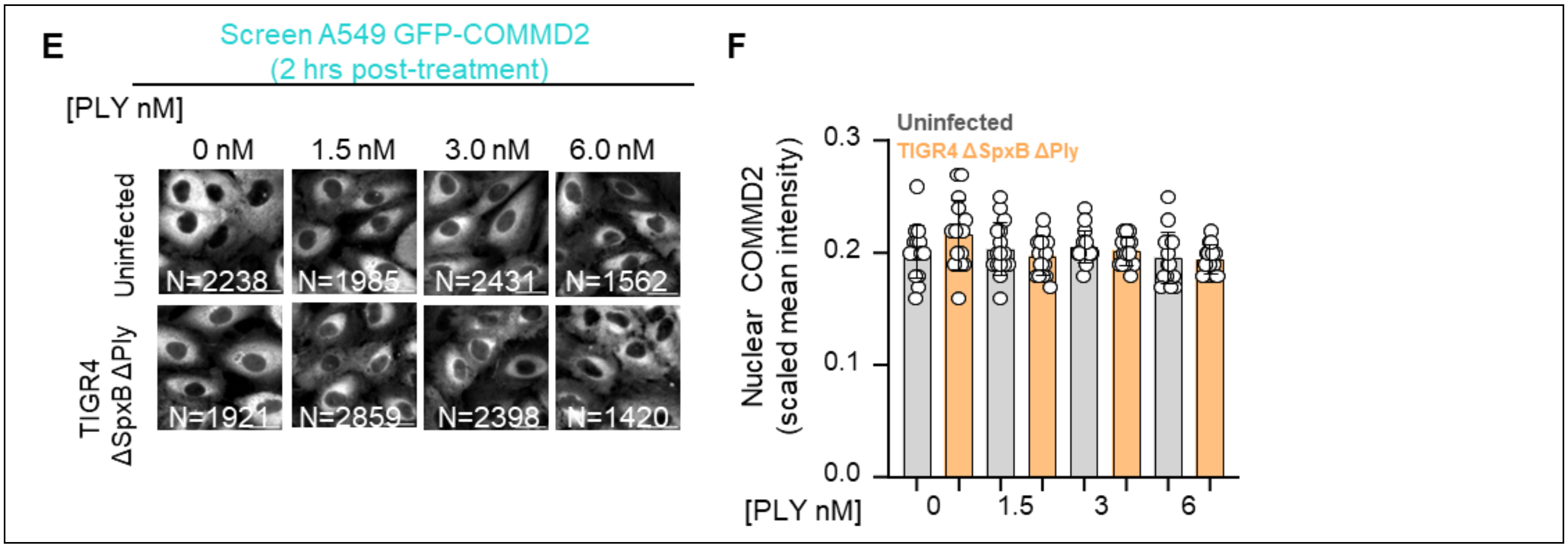

10. The same is true for SpxB and the production of hydrogen peroxide; or does the data mean there is growth of TIGR4 but not serotype 6B in the infection medium?

We have now deeply addressed this matter in the new manuscript Fig 8 and Sup. Fig 10, where we do titration of hydrogen peroxide against several pneumococcal mutants to clearly show it is intrinsic on the synergistic effect of both bacterial virulence associated proteins.

Additionally, we did test CFU and hydrogen peroxide levels after both 2hrs of bacterial challenge of A549 cells and in midlog growth media across multiple pneumococcal strains favoring commensal-like or invasive host outcomes (data below) using Amplex™ Red Peroxidase/Hydrogen Peroxide Assay Kit (Thermo cat# A22188). The below data is not included within the manuscript.

Our results demonstrate that after 2hr A549 challenge, using matched CFU inocula, there is no biological difference in bacterial growth or hydrogen peroxide production (A – C). For this experiment matched inocula of each strain was used to challenge A549 cells for 2 hrs, with the supernatant (200ul) in triplicate collected, centrifuged to remove bacteria, and 100ul (in triplicate) moved to a clear flat bottom 96well plate for Amplex Red testing. While the pneumococcal strain 1 ST304 shows a statistical difference in comparison to TIGR4, we believe that to be an artifact of statistical testing and not a reflection of biology (C). We repeated this experiment 4 biological times.

In a complementary test, as the presence of host cells could alter expression pneumococcal expression of SpxB and thus hydrogen peroxide production, we tested the levels of hydrogen peroxide in midlog bacterial growth media. For this 1mL of culture at 0.6 OD_{600} was collected via centrifugation to pellet bacteria, then 100ul aliquots (triplicate) moved to a clear flat bottom 96well plate for Amplex Red testing. There was no statistical difference in bacterial numbers or the amount of hydrogen peroxide. We repeated this experiment 4 biological times.

Of note, there is roughly a 9-fold reduction in hydrogen peroxide amount between bacterial growth culture and collected cell supernatants.

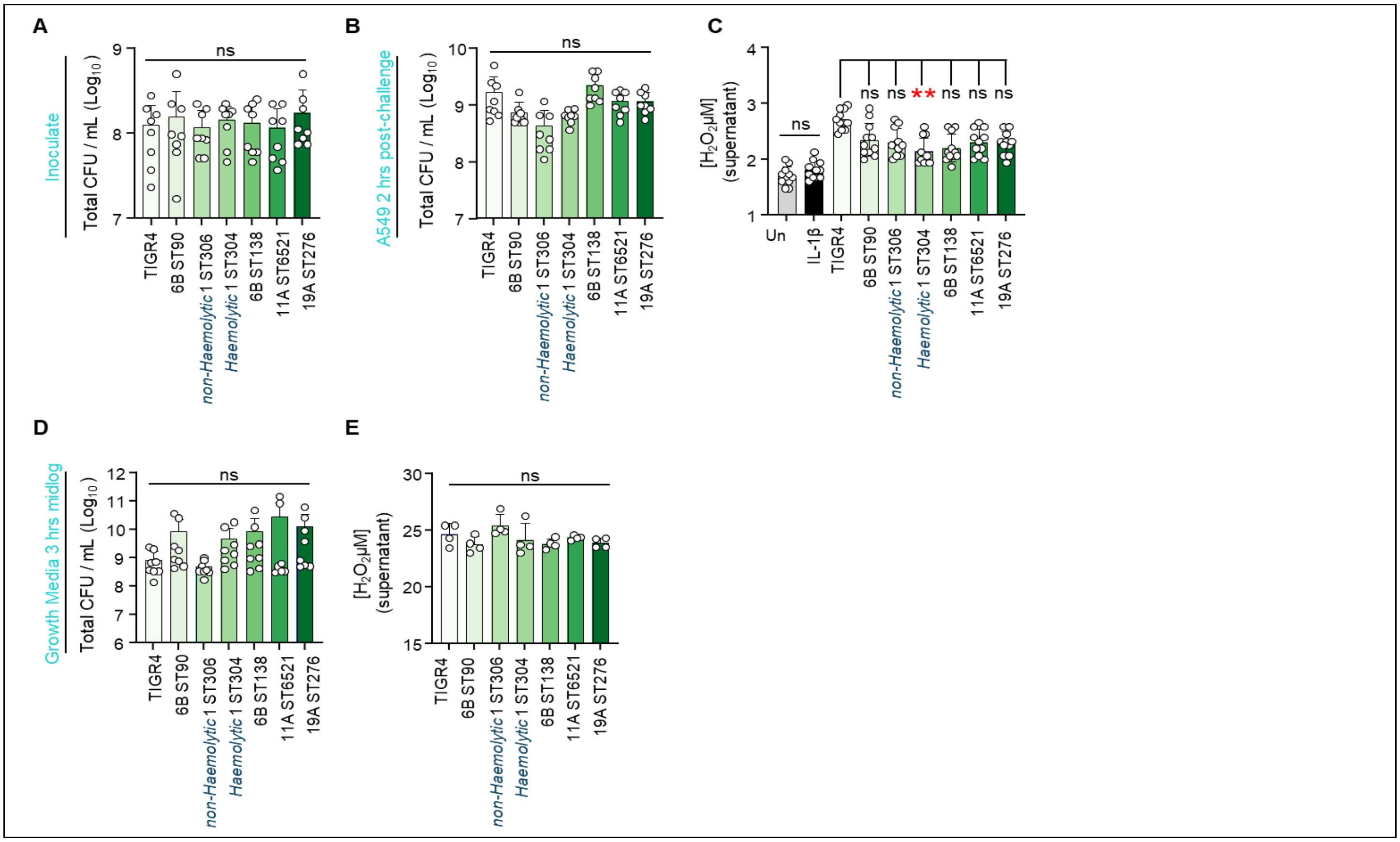

11. Can the effect of pneumolysin under the selected conditions been inhibited e.g. by cholesterol or specific antibodies?

This is an interesting comment; one we have given considerable thought to. We believe these direct experiments are outside the focus of the current manuscript.

However, our in-depth work in Fig 8 and Sup. Fig 10 leading to the discovery of association for COMMD2 translocation to invasive potential of pneumococcus lays a future framework for deeper investigation with a neutralizing PLY antibody, or exogenously/chemically depleting/adding cholesterol during infection to identify novel virulence associated bacterial proteins.

Reviewer #1 (Remarks to the Author):
The manuscript is much improved. A few issues remain, which need to be resolved. They are not critical to the key conclusions of the paper so I believe that this should be addressable prior to publication:

We thank you for these comments and acknowledgement of our efforts.

- The images for nulear localization of phospho-RelA-S536 in untreated cells are confusing, as they seem to indicate contitutive nuclear localization at high levels which do not match the graph shown. Please review and amend or explain.

We have now clarified this point. Indeed, indicated next to each phospho-RelA-S536 image is the LUT intensity scale, which is different for each sample. We have added text to explain this better in lines 156-165.

- The WB blot to demonstrate the level of expression of GFP-COMMD2 vs the endogenous protein should be shown in the paper and should contain in the same membrane samples from the parental line and the GFP-COMMD2 line. Showing two separate membranes in one panel does not address whether the line expresses physiologic levels of COMMD2.

Thank you for this suggestion. We have moved the blots into the manuscript as part of the Sup. Fig 8 (B & C) and have added corresponding lines in the text (Lines 280-291). The crops of the blot in the rebuttal depict 2 biological replicates for each cell type from the same whole PVDF membrane. The full blots have been added into the "Supplemental materials Immunoblots". We have also added the raw data for the ratios into the "Sup. Table 4 Figure_Data".

- In Fig S6, there is a big difference in the mobility of COMMD2 between input and IP lanes which suggests that the band in the IP is not specific. This experiment is problematic as presented.

We appreciate the reviewer's comment on the weight shift observed in this endogenous immunoprecipitation blot. We had specifically commented on this in in lines 304 – 307 when we report our proteomics findings. We believe this shift in kda is in-line with the addition of a ubiquitin moiety (8.6kDa) to the immunoprecipitation blot using native p65. Correlated to this are the results in which we identify an enriched ubiquitination at the COMMD2 Lysine residue 50 (Sup. Table 2). Taken together we do not believe this to be a non-specific band as the western blot is clean of other bands.

- The siRNA experiments knocking down COMMD2 produce very modest to minimal knowckdown and therefore are technically problematic.

We added in our revised manuscript a second siRNA targeting COMMD2, with the ID# s27490 (C-terminal) generating slightly better knockdown than the first (ID# s27488; N-terminal) which was ~40-50% knockdown. This decrease might be considered minimal by the reviewer, but even with this level of knockdown, we significantly reduced COMMD2 levels with the second siRNA in comparison to the scrambled control. For full transparency to the reader, we acknowledge the limitation of this experiment in lines 317 – 320. However, overall, we do not find this is problematic as this modest reduction still resulted in differences in analyte expression.
